# The rise and demise of the Paleogene Central Tibetan Valley

**DOI:** 10.1126/sciadv.abj0944

**Published:** 2022-02-09

**Authors:** Zhongyu Xiong, Xiaohui Liu, Lin Ding, Alex Farnsworth, Robert A. Spicer, Qiang Xu, Paul Valdes, Songlin He, Deng Zeng, Chao Wang, Zhenyu Li, Xudong Guo, Tao Su, Chenyuan Zhao, Houqi Wang, Yahui Yue

**Affiliations:** 1 State Key Laboratory of Tibetan Plateau Earth System, Environment and Resources (TPESER), Institute of Tibetan Plateau Research, Chinese Academy of Sciences, Beijing 100101, China.; 2 University of Chinese Academy of Sciences, Beijing 100049, China.; 3 School of Geographical Sciences, University of Bristol, Bristol BS8 1SS, UK.; 4 CAS Key Laboratory of Tropical Forest Ecology, Xishuangbanna Tropical Botanical Garden, Chinese Academy of Sciences, Mengla 666303, China.; 5 School of Environment, Earth and Ecosystem Sciences, The Open University, Milton Keynes MK7 6AA, UK.

## Abstract

Reconstructing the Paleogene topography and climate of central Tibet informs understanding of collisional tectonic mechanisms and their links to climate and biodiversity. Radiometric dates of volcanic/sedimentary rocks and paleotemperatures based on clumped isotopes within ancient soil carbonate nodules from the Lunpola Basin, part of an east-west trending band of basins in central Tibet and now at 4.7 km, suggest that the basin rose from <2.0 km at 50 to 38 million years (Ma) to >4.0 km by 29 Ma. The height change is quantified using the rates at which wet-bulb temperatures (*T*_w_) decline at land surfaces as those surface rise. In this case, *T*_w_ fell from ~8°C at ~38 Ma to ~1°C at 29 Ma, suggesting at least ~2.0 km of surface uplift in ~10 Ma under warm Eocene to Oligocene conditions. These results confirm that a Paleogene Central Tibetan Valley transformed to a plateau before the Neogene.

## Teaser

Clumped isotope and climate model shows that central Tibet rose rapidly from a low valley to form a high plateau by 29 Ma.

## INTRODUCTION

Accurate quantification of how the modern Himalaya and Tibetan Plateau came to form Earth’s most notable elevated feature is critical to assess its influence on atmospheric (e.g., monsoons and large-scale circulation) and land surface processes (e.g., vegetation characteristics, sedimentation, and erosion rates) (*1*–*4*). Ideas concerning the growth of this complex orogenic system have evolved from considering a simultaneous rise of the entire Tibetan region as a single entity through to differential surface height changes (*5*–*9*), but the exact patterns and related geodynamic processes of topographic development remain in debate. Past concepts suggested that the first parts of the region to reach high elevations were in southern Tibet and that they did so in the Eocene. Subsequently, this rise propagated northward as India indented deep into Asia (*9*–*12*). Another view envisaged that the Eocene central Tibet was the most elevated part of the region, which formed a high proto-Tibetan Plateau that then expanded southward to form the Himalaya and northward to elevate northern Tibet, primarily from the Miocene onward (*8*, *13*, *14*).

**Fig. 1.**
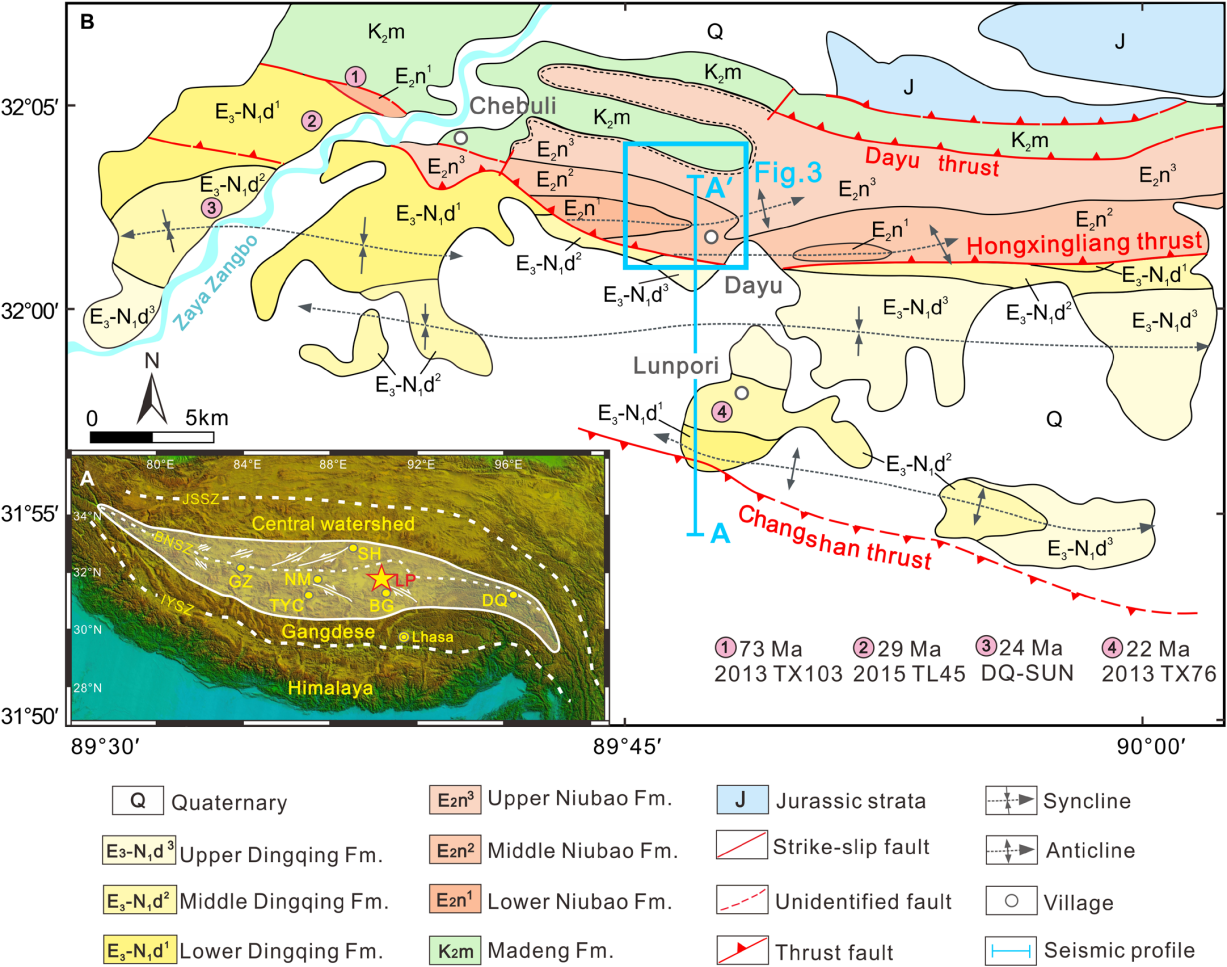
Geomorphological and geological maps showing the location of the Lunpola region and the measured sections. (**A**) Topography of the Tibetan Plateau and neighboring regions illustrating the main tectonic boundaries, the extent of the Central Tibetan Valley (white shaded area), V-shaped conjugate strike-slip faults (*22*–*23*), the location of the Lunpola Basin (yellow star), and other Cenozoic basins (yellow circles). (**B**) Geologic map of the Lunpola Basin. The blue line indicates the position of the seismic profile (A-A′) in Fig. 2. The sample locations for U-Pb dating near Chebuli and Lunpori sections are shown with pink circles. The blue rectangle indicates the mapped Dayu area in Fig. 3, and the sample locations and U-Pb dating results at the Dayu section are detailed in Figs. 3 and 4. BG, Bangor Basin; DQ, Dingqing Basin; GZ, Gerze Basin; IYSZ, Indus-Yarlung Suture Zone; JSSZ, Jinsha Suture Zone; LP, Lunpola Basin; NM, Nima Basin; SH, Shuanghu Basin; TYC; Tangra Yum Co Basin.

Recent studies point to a complex Paleogene topography predating plateau formation, with two high mountain systems (the Gangdese and Central Watershed mountains, the latter including the Tanghula or Tanghla Mountains) sandwiching central Tibetan lowlands (*7*, *15*–*17*), and we term these lowlands the “Central Tibetan Valley.” We argue that this valley extended along most of the length of the ancient junction between two of the major allochthonous tectonic blocks, the Lhasa and Qiangtang terranes, that collided in the Mesozoic and today form the core of the plateau (*18*–*20*). This topographic depression along the Banggong (Bangong)-Nujiang Suture Zone (BNSZ) is today terminated by a series of V-shaped conjugate strike-slip faults where, in surface view, triangular extensional basins are visible [Fig. 1A; (*21*, *22*)]. The present high-elevation low-relief landform extends east-west over a distance of ~1500 km and is 20 to 100 km wide in a north-south direction (Fig. 1A), but ample geological evidence points to significant (>50%) South to North (S-N) shortening throughout its length during the Paleogene (*14*, *23*). Given the progressive compression from India, this remnant of the ancient Central Tibetan Valley undoubtedly narrowed over time, but how it changed in width and depth throughout the Paleogene is not yet quantified. The history of the valley is now archived in a line of sedimentary basins within the BNSZ, with the Dingqing Basin in the east; the Bangor, Lunpola, and Nima Basins in the middle; and the Gerze Basin in the west (Fig. 1A).

Several paleoaltimetric approaches based on carbon, hydrogen, and oxygen isotopes, as well as animal and plant fossils, have been used to explore changes in the heights of the basins along the Central Tibetan Valley (*16*, *17*, *24*–*30*), but the outcomes lack consensus. Foraminifera found in the Gerze Basin toward the western end of the ancient valley suggest that the basin was near the sea level in the Eocene (*24*), whereas, in the Lunpola Basin, oxygen isotopes from paleosol carbonate and hydrogen isotopes from lipid biomarkers within the Niubao and Dingqing formations have been interpreted to suggest that the Lunpola area has maintained a high elevation (~4.5 km) since the Late Eocene (*25*, *26*). However, estimates from palynological and mammalian fossils preserved within the Dingqing Formation suggest an elevation of ~3.0 km during Late Oligocene-Miocene interval (*27*, *28*), which is consistent with a leaf wax lipid carbon-oxygen isotopes estimate (*29*). Megafossils, including leaf and fish fossils, were previously reported to be from the Dingqing Formation and thought to have been deposited at ~25 million years (Ma) (*17*, *30*), but recent dating of what appears to be a primary tuff associated with the fossils shows that they are Late Eocene in age (~38 Ma) and belong to the Niubao Formation (*31*). Consequently, the earlier paleoelevation estimate based on them, which limited the basin elevation to lower than 2.3 km before the Late Oligocene (*17*), could be too low. This is because the Late Eocene was warmer than the Late Oligocene (*32*) and so the temperature-sensitive palm fossils upon which the height estimate was based might have survived at greater elevations. Underlying fossil assemblages from the Middle Niubao Formation in the nearby Bangor Basin are dated to the Middle Eocene (but no older than 47 Ma) based on zircons in tuffaceous units and, using moist enthalpy and conservation of energy principles, quantify the elevation of the basin floor to have been 1.5 ± 0.9 km (*16*).

The lack of an absolute chronological framework throughout the Cenozoic stratigraphy of the Central Tibetan Valley and the diversity of paleoelevation estimations have become important factors at the heart of the arguments surrounding the Paleogene topography of Tibet and exactly when it transformed into the high, but low relief, surface that we see today.

**Fig. 2.**
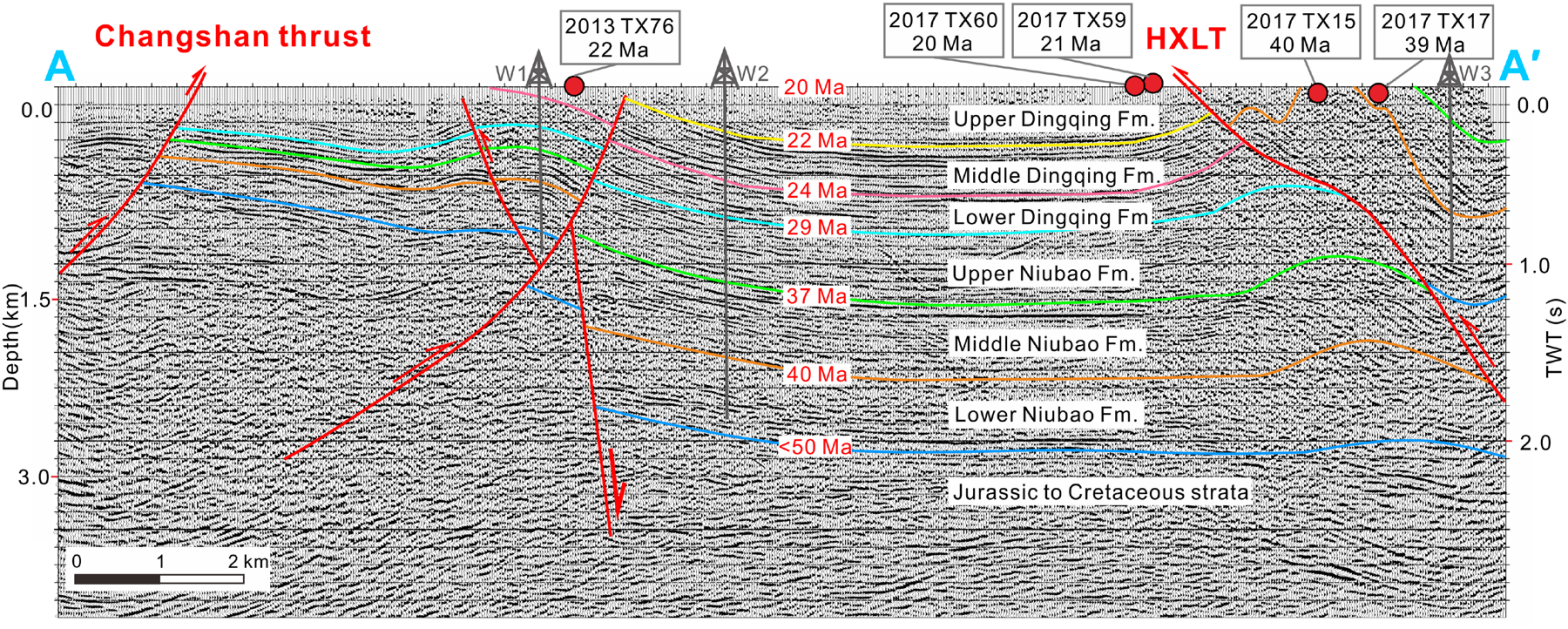
Stratigraphic geochronology of the Lunpola Basin. Interpretation of the seismic profile along the section A-A′ (*57*) shown in Fig. 1B. The classification of strata, thrusts, and U-Pb ages (denoted by filled red circles) of the Niubao and Dingqing formations in the Dayu section are expressed along the profile. Three wells used for layer calibration are denoted as W1 to W3. HXLT, Hongxingliang thrust.

The Lunpola Basin sedimentary record, at a present elevation of ~4.7 km, has the potential to address dating and paleoelevation issues. The basin is one of the several remnant depositional centers that accumulated Cenozoic sediments along the BNSZ (Fig. 1) (*18*) and hosts the most complete Cenozoic sedimentary succession in central Tibet with a total thickness of 3 to 4 km, comprising the Niubao and Dingqing formations (Figs. 1B and 2).

To help resolve the elevation history of the Central Tibetan Valley, we first provide a more secure chronostratigraphy than has hitherto been available. This age regime is based on radiometric dating throughout the Cenozoic succession in the Lunpola Basin. Recent magnetostratigraphic dating of the upper part of the Lunpola succession exposed in the Dayu section (*31*) is questioned here because it is contradicted by our radiometric dates. Second, we introduce a new method of translating soil temperatures, as measured by carbonate-clumped isotopes, into surface elevation. We do this by using model-generated estimates of how past temperature changed with height at Earth’s surface (known as terrestrial thermal lapse rates) and not, as is usually used, vertically in the free atmosphere (free air or so called “environmental,” thermal lapse rates). We explore uncertainties in this approach by using a range of different topographic scenarios (*33*). Moreover, we use wet-bulb instead of the more conventional dry-bulb temperatures because wet-bulb temperatures accommodate inevitable changes in humidity within a parcel of air as it passes over land surfaces of varying heights (*33*).

## RESULTS

### Stratigraphic geochronology and structural evolution

Field mapping indicates that the Cenozoic strata of the Lunpola Basin are bounded by several east-west trending, basin-scale thrust faults (Fig. 1B) that demonstrate S-N crustal shortening or compression processes. The southern boundary of the basin is marked by the regionally extensive, southward-dipping Changshan thrust with a Jurassic-Cretaceous complex in the hanging wall, while the Dingqing Formation is in the foot wall (Fig. 1B). The northern boundary is deformed by the northward-dipping Dayu thrust with the Jurassic complex and Cretaceous volcanics in the hanging walls, while the Niubao Formation occurs in the foot wall (Fig. 1B).

**Fig. 3.**
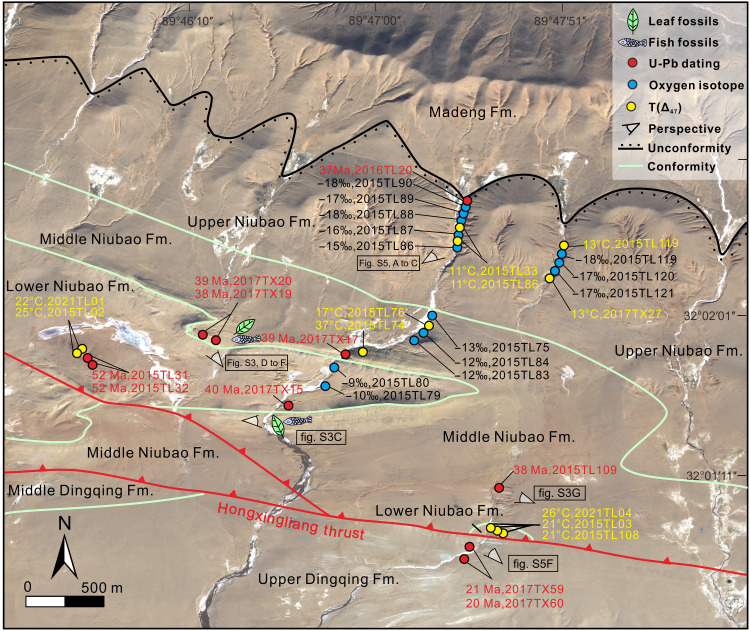
Map of the Dayu area. Strata classification, structure, U-Pb ages, carbonate oxygen isotope (δ^18^O_c_), and clumped isotope–derived temperature [*T* (∆_47_)] information superimposed on a high-resolution satellite picture from Landsat 8.

The Hongxingliang thrust passes through the basin center and places the Niubao Formation onto the younger Dingqing Formation near the Dayu section (Fig. 1B). Seismic profiles (Fig. 2) and high-resolution remote sensing satellite imagery (Landsat 8; Fig. 3) show that the Niubao Formation was deformed as eastward plunging fault-propagation folds in the hanging wall, while the Dingqing Formation was deformed as several subdued wavelength folds in the footwall. Because of the southward thrusting of the Hongxingliang thrust, the upper member of the Niubao Formation is eroded and not exposed in the southern limb of the Dayu anticline, while the middle member of the Niubao Formation is exposed on both limbs of the anticline (Fig. 3). The thrusting and folding in the Niubao Formation resulted in close contact of the middle member of the Niubao Formation with the upper member of the Dingqing Formation (Figs. 2 and 3). The similar colors and lithofacies of the middle member of the Niubao Formation with the upper member of the Dingqing Formation (Fig. 3) become an obstacle in differentiating these two units correctly in the field.

**Fig. 4.**
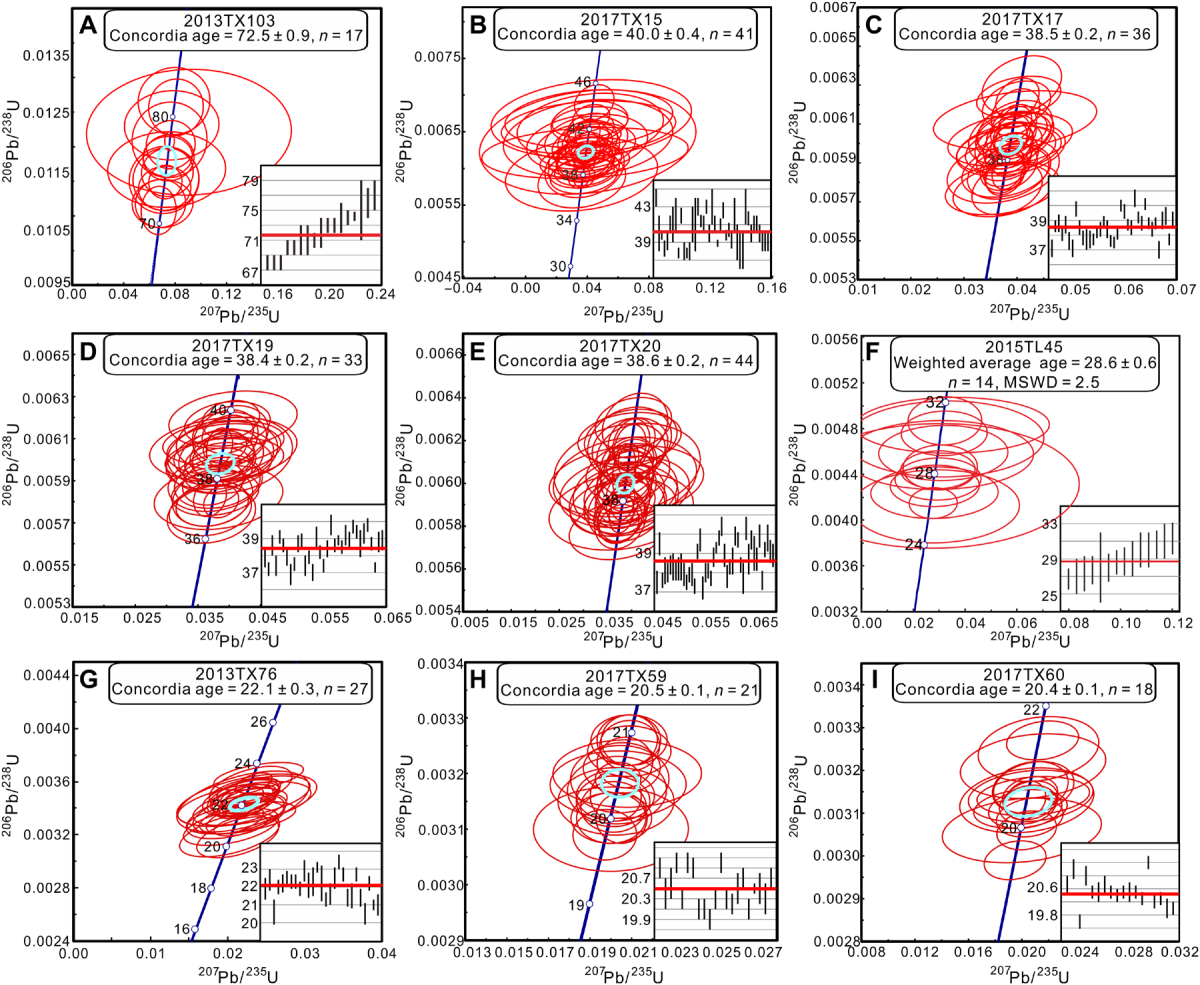
Zircon U-Pb ages of volcanic tuffs from the Lunpola Basin. Light blue circles indicate the Concordia ages of each sample. (**A**) Volcanic rock from the Cretaceous Madeng Formation. (**B**) Tuff from the upper part of the lower member of the Niubao Formation. (**C** to **E**) Tuffs from the middle member of the Niubao Formation. (**F**) Tuff from the lower member of the Dingqing Formation. MSWD, mean square weighted deviation. (**G**) Tuff from the middle member of the Dingqing Formation. (**H** and **I**) Tuffs from the upper member of the Dingqing Formation.

With a clear structural background for the Lunpola Basin, we measured four stratigraphic sections (Chebuli, Dayu, Dawei, and Lunpori) (figs. S1and S2; see details in the Supplementary Materials) and combined their stratigraphies with U-Pb geochronology to show that the Niubao Formation, with thickness of 2 to 3 km in the central basin (Fig. 2), unconformably overlies the Late Cretaceous Madeng volcanics (73 Ma; Fig. 4A and figs. S2A and S3A) and can be divided into three (lower, middle, and upper) members.

The lower member of the Niubao Formation is characterized by purple-red to yellow clastic deposits, comprising mostly conglomerates and sandstones with several paleosols developed in situ (fig. S3, A and B). For any given sandstone, the three youngest detrital zircon grains yielding ^206^Pb/^238^U ages that overlap with their mean at 2σ constrain the maximum depositional age of that sandstone (*34*). The youngest three zircons of sandstone (2013TX53) from the lower member of the Niubao Formation at the Chebuli section yield a weighted mean age of 48.8 ± 2.6 Ma (2σ; figs. S2A and S4A), while sandstones (2015TL31-32) from the Dayu section yield weighted mean ages of 52.2 ± 2.6 Ma and 52.3 ± 2.7 Ma (2σ; figs. S4, B and C), respectively. Therefore, the maximum depositional age of the lower member of the Niubao Formation is constrained to ~50 Ma. One tuff sample (2017TX15) found in the upper part of this member, and exposed in the same section, defines the youngest age of the unit and yields a Concordia age of 40.0 ± 0.4 Ma (2σ, *n* = 41; Fig. 4B). Given the stratigraphic relationships and zircon ages of the tuff layers and sandstones, the depositional age of the lower member of the Niubao Formation is constrained to ~50 to 40 Ma.

The middle member of the Niubao Formation consists of alternating grayish-green to grayish-white mudstones, marls, tuffs, and sandstones with leaf and fish fossils (Fig. 3 and figs. S2, B and C, and S3, C to G) previously attributed to the lower member of the Dingqing Formation (*17*, *30*). Three tuff samples (2017TX17, 2017TX19, and 2017TX20; fig. S3, C to F) have been found within the middle member in the Dayu section and yield Concordia ages of 38.5 ± 0.2 Ma (2σ, *n* = 36; Fig. 4C), 38.4 ± 0.2 Ma (2σ, *n* = 33; Fig. 4D), and 38.6 ± 0.2 Ma (2σ, *n* = 44; Fig. 4E), respectively. The youngest zircon age from the sandstones in the upper part of the member is 38.2 ± 1.2 Ma (2σ; figs. S2C, S3G, and S4D) and found at the Dawei section. The middle member of the Niubao Formation is therefore constrained to range in age from 40 to 38 Ma.

The upper member of the Niubao Formation is composed of red-brown clastic deposits, mainly including conglomerates and sandstones with several in situ developed paleosols (figs. S2, B and C, and S5, A to C). The youngest zircon age from sandstones found in the uppermost part of the paleosol is 37.3 ± 1.6 Ma (2σ; figs. S4E and S5A), which limits the maximum depositional age of the member, and the upper limit of the member is conservatively constrained by the zircon weighted average age (28.6 ± 0.6 Ma, 2σ, *n* = 14; Fig. 4F) from an overlying tuff layer (fig. S5D) in the lower member of the Dingqing Formation at the Chebuli section. Therefore, the depositional age range of the upper member of the Niubao Formation is constrained to 37 to 29 Ma.

The Dingqing Formation, with a thickness of ~1 km in the central basin, overlies the Niubao Formation and is also divided into the lower, middle, and upper members (Fig. 2). The lower member of the Dingqing Formation consists of gray to yellowish-gray mudstones, shales, marls, and fine-grained sandstones with tuffite layers (fig. S5D). Combined with a previously U-Pb–dated bentonite with an age of 23.6 ± 0.2 Ma (*35*) from the upper part of the lower member of the Dingqing Formation, the depositional age is constrained to 29 to 24 Ma. The middle member of the Dingqing Formation consists of gray mudstones, shales, and oil shales interbedded with siltstones and fine-grained sandstones with tuffite layers (fig. S5E). The age of this member is constrained to be ~24 to 22 Ma by the U-Pb Concordia age of 22.1 ± 0.3 Ma (2σ, *n* = 27; Fig. 4G) from a tuff layer found at the Lunbori section. The upper member of the Dingqing Formation consists of gray mudstones and shales, maroon mudstone interbedded with siltstones, and tuffite layers (fig. S5F). The age of the upper member is constrained to be ~22 to 20 Ma by U-Pb Concordia ages (20.5 ± 0.1 Ma, 2σ, *n* = 21; Fig. 4H) and 20.4 ± 0.1 Ma, 2σ, *n* = 18; Fig. 4I) from two tuff samples found near the Dayu section.

Our detailed field mapping, seismic interpretation, and nine U-Pb Concordia ages from tuff zircons here establish an absolute chronological framework for the Cenozoic strata (the Niubao and Dingqing formations) in the Lunpola Basin and confirm the ~38 Ma age of the Dayu fossil assemblage reported previously (*17*, *30*, *31*). The depositional age range of the upper member of the Niubao Formation is here determined to be 37 to 29 Ma and the lower member of the Dingqing Formation to be 29 to 24 Ma. These absolute dates and seismic data lead us to reject the paleomagnetostratigraphic framework as proposed in (*31*) because (i) the age of the overlying lower member of the Dingqing Formation (29 to 24 Ma) constrained by our radiometric dates overlaps the inferred age (27 to 23 Ma) in (*31*) for the upper member of the Niubao Formation; (ii) the true thickness of the middle member of the Niubao Formation is ~0.7 km as evidenced by seismic profile (Fig. 2), while the paleomagnetostratigraphic column of (*31*) shows 1.3 km and thus may indicate repeat measurements; and (iii) several erosion surfaces at the bases of conglomerates, and periods of nondeposition in the form of paleosols, are evident in the upper member of the Niubao Formation.

### Eocene to Oligocene topographic rise of central Tibet

A combined proxy approach for determining past elevations is more robust than any based on a single line of evidence. Surface temperature and oxygen isotope values (δ^18^O) of paleosol carbonates and of paleo-precipitation decrease systematically with elevation and, within an accurate chronology, make them potentially useful paleoaltimeters (*25*, *36*, *37*). Here, we provide two independent constraints on surface heights by (i) converting carbonate formation temperatures [*T* (∆_47_)] in soil to wet-bulb land surface air temperatures and then estimating elevation using model-derived wet-bulb terrestrial lapse rates and (ii) measuring the decrease in oxygen isotope of paleo-surface water (δ^18^O_p_) derived from precipitation.

**Fig. 5.**
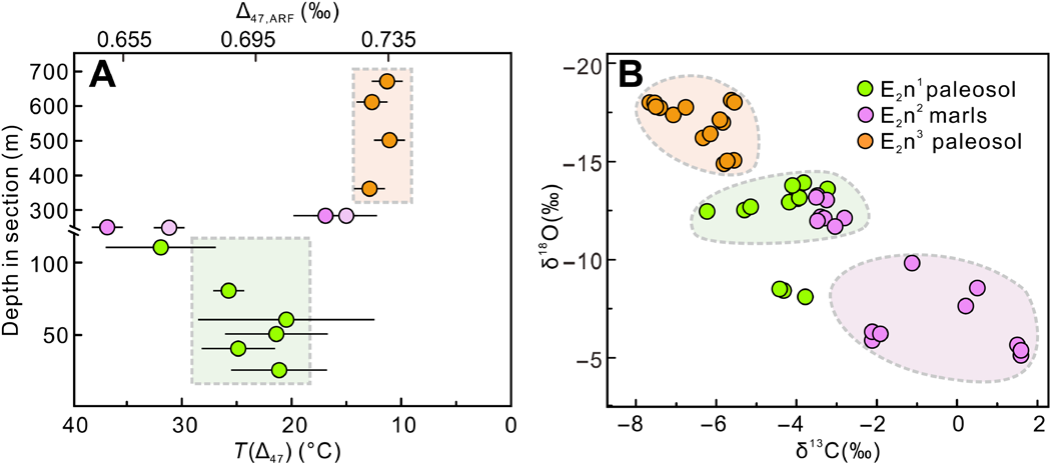
Clumped, carbon, and oxygen isotope results from the Lunpola Basin. (**A**) *T* (Δ_47_) of samples from the three members of the Niubao Formation. The *T* (Δ_47_) decreases ~12°C from the lower (E_2_n^1^) to the upper (E_2_n^3^) part of the Niubao Formation. Filled light purple circles denote the calculated lake summer temperature. ARF, absolute reference frame (*63*). (**B**) Carbon and oxygen isotopes from carbonates in the Niubao Formation. The δ^18^O from the upper member of the Niubao Formation shows −4‰ decrease when compared with the lower member of the Niubao Formation, while the middle member of the Niubao Formation shows scattered δ^18^O values ranging from −13.2 to −5.2%.

The Δ_47_ value of six carbonate nodules from the lower member of the Niubao Formation ranges from 0.669 to 0.705 per mil (‰) with an average value of 0.694 ± 0.019‰ (1σ; Fig. 5A), and four carbonate nodules from the upper member of the Niubao Formation have Δ_47_ values that range from 0.731 to 0.737‰, with an average value of 0.734 ± 0.010‰ (1σ; Fig. 5A). Two lacustrine marls from the lower and upper part of the middle member of the Niubao Formation have Δ_47_ values of 0.653 ± 0.010‰ (1σ) and 0.717 ± 0.012‰ (1σ; Fig. 5A), respectively. The Δ_47_ is converted into temperature via the empirical thermometer in (*38*). The calibrated *T* (∆_47_)s from the lower member of the Niubao Formation range from 20.5° to 32°C with an average value of 23.9° ± 5.9°C (1σ; Fig. 5A). The *T* (∆_47_) from the upper member of the Niubao Formation ranges from 10.1° to 12.9°C with an average value of 11.9° ± 2.8°C (1σ; Fig. 5A). Two lacustrine marls have *T* (∆_47_)s of 37.4° ± 2.8°C and 17.0° ± 3.4°C (Fig. 5A). The transformation of the *T* (∆_47_) of marls to lake summer temperatures using an empirical relationship derived from modern lacustrine authigenic carbonates in Western China (*39*) suggests a decrease by 15°C from 31.2° to 15.3°C (Fig. 5A), which is comparable with the *T* (∆_47_) difference from the lower to upper members of the Niubao Formation.

The oxygen isotope (δ^18^O) values of the paleosol carbonates from the lower and upper members of the Niubao Formation yield an average value of −13.2 ± 0.5‰ (1σ; Fig. 5B) and −17.0 ± 1.1‰ (1σ; Fig. 5B), respectively, and thus show a −4‰ decrease moving upsection. The marls from the middle member of the Niubao Formation show scattered δ^18^O values, varying from −13.2 to −5.2‰ (Fig. 5B), which indicate lake evaporation effects (*40*).

### Interpreting soil temperatures

**Fig. 6.**
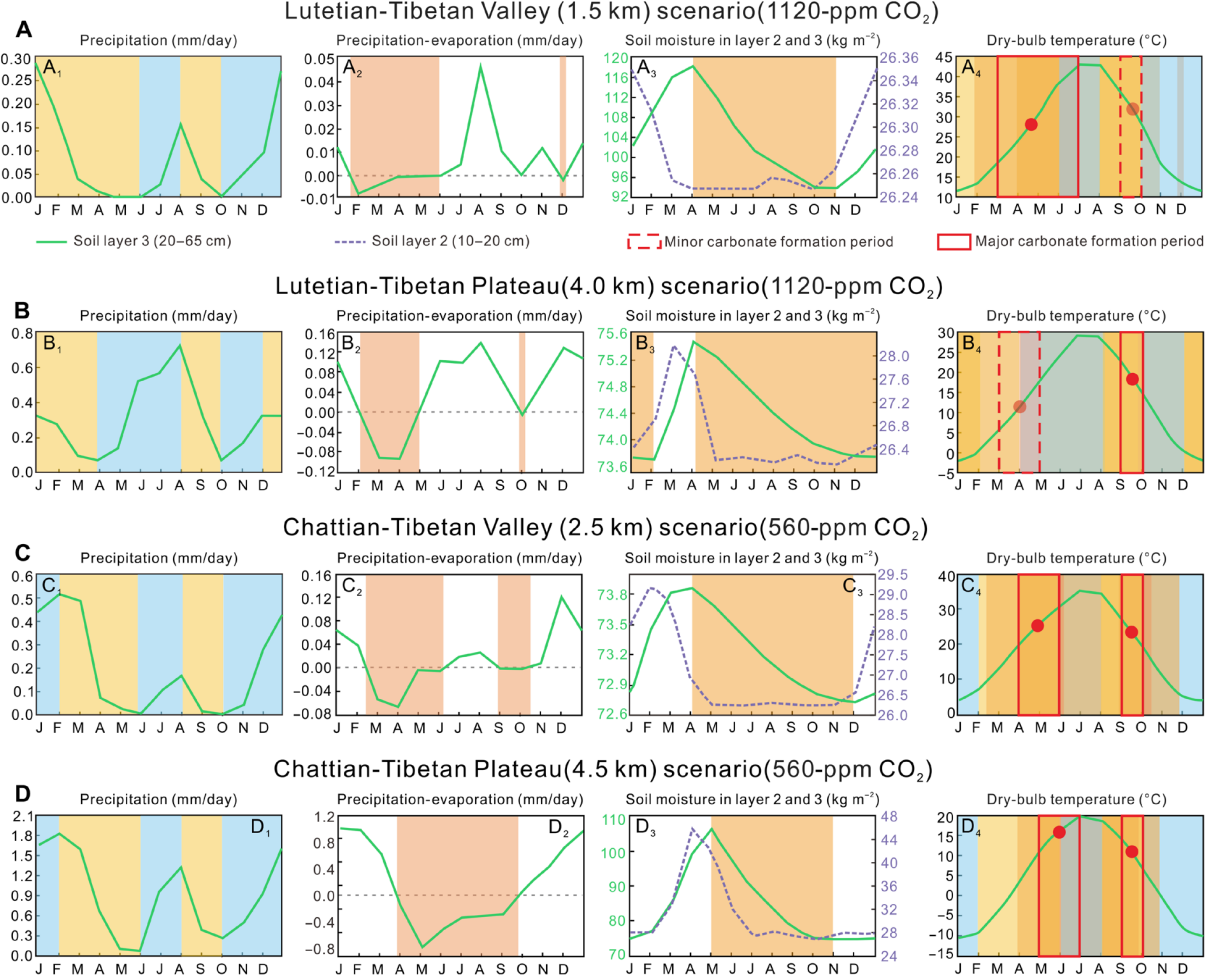
Modeled precipitation, precipitation-evaporation, soil moisture, and temperature regimes (note different scales) for central Tibet for different orographic scenarios and atmospheric compositions. Evaporation is controlled by the canopy conductance term, which in turn is calculated by the temperature, humidity deficit, incident radiation, soil moisture availability, and vegetation type in the model. Soil drying is a function of both evaporative processes and transpiration from model-generated vegetation. The plots are primarily for soil layer 3 (between 20 and 65 cm depth), but, for soil moisture, we have shown results for the soil layer 2 (10- to 20-cm depth). Layer 2 shows more dynamic wetting and drying than at greater depths and informs interpreting when carbonate nodule form may have occurred in soil layer 3. (**A**) Lutetian valley sets at 1.5 km bounded by the Gangdese and Central Watershed mountains set at 4.5 and 4.0 km, respectively, and kept stable in scenario 2. Atmospheric CO_2_ set at 1120 ppm. Drying conditions are shown by yellow, pink, and orange shading, while blue shading represents periods of increasing precipitation (A_1_ to A_4_). The different intensities of shading in the dry-bulb temperature plots (A_4_) indicate the overlaps of shading shown in the precipitation (A_1_), evaporation (A_2_), and soil moisture (A_3_) plots. The most likely carbonate-forming periods (in monthly units) are indicated on the temperature plots with red rectangles, with less likely periods shown by rectangles with dotted margins (see text for detailed reasoning). Red-filled circles indicate monthly average dry-bulb temperatures (*T*_d_) near surface during those nodule-forming periods as predicted by the water isotope–enabled climate model. The same explanations apply to (**B**) to (**D**). (B) Lutetian plateau is set at 4.0 km and CO_2_ is set as in scenario 1. (C) Chattian valley floor is set at 2.5 km, the Gangdese and Central Watershed mountains are at 5.0 km. CO_2_ concentration is set at 560 ppm. (D) Chattian plateau is set at 4.5 km.

Transforming *T* (∆_47_) into a surface height is not straightforward. Soil depth and texture, vegetation, the seasonal precipitation pattern, and *p*CO_2_ will all influence the formation of paleosol nodules and result in seasonal biases in *T* (∆_47_) values (*41*–*44*). The time(s) of the year when the carbonate nodules form in paleosols is critical in the interpretation of *T* (∆_47_), and the relationship between *T* (∆_47_) and surface air temperature will further influence the extended application of *T* (∆_47_) as a paleoclimate and paleoelevation archive. Prior work has made empirical assumptions that soil carbonate formation will be biased toward the summer season when soils are drying [but not dry; (*42*)], and temperatures have been interpreted accordingly with scant regard to the actual precipitation and soil drying regimes that existed in the past. Here, we use the model-predicted climate, including soil moisture, to better constrain times of potential soil carbonate formation (Fig. 6).

The soil carbonates were collected from paleosols with a silt to sand matrix component (fig. S6) and so likely dehydrated soon after precipitation events (*41*). The sampling depth of nodules was below 40 cm from the top of the paleosols, but we recognize that this depth does not accommodate compaction and erosion after the initial soil formation, so the actual formation depth may have been below this. At such a depth, it is reasonable to assume nodule formation will be biased toward soil drying following large precipitation events typical of the rainy season rather than immediately after small frequent precipitation events throughout the year (*43*). Figure 6 shows the drying regime for the model soils at 10 to 20 cm depth (soil layer 2) and 20 to 65 cm depth (soil layer 3), respectively. At shallower depths, soil moisture responds more rapidly to precipitation events but also dries more quickly.

To determine when in the year nodule formation was most likely, we examined model-generated precipitation, propensity for drying (precipitation minus evaporation), and soil moisture at two soil depth ranges (10 to 20 cm and 20 to 65 cm), which encompasses our sampling depth within the paleosols. We did this for valley and plateau scenarios for Lutetian and Chattian boundary conditions (fig. S7).

Figure 6A shows that, for a central Tibet Lutetian valley floor set at 1.5 km and 1120-ppm (parts per million) *p*CO_2_ levels, carbonate nodules were most likely to form during March to June and again in September. Note that the annual precipitation is bimodal with peak precipitation in the winter and lesser amounts in summer, but that drying conditions become established quickly at shallow soil depths (soil layer 2) as precipitation declines and evaporation exceeds precipitation toward the end of January (Fig. 6, A_1_ to A_3_). By April, soil moisture also starts to decline deeper in the soil profile (layer 3), but, as indicated by conditions in layer 2, drying near the top of layer 3 would have been established sooner than at 65 cm (Fig. 6A_3_). At nodule sampling depth (~40 cm or greater), drying would have begun in March, and summer rains only lessened the drying rate but did not halt it. Nevertheless, the additional water percolating through the soil profile in the summer rainy season may have curtailed carbonate formation and may even have resulted in dissolution, so we assume the bulk of carbonate formation only extended through June (Fig. 6A_4_). At nodule depth, a second minor episode of carbonate formation may have occurred in September as the soil dried after the summer rains, but the most significant carbonate formation took place in the late spring to early summer (March to June) based on a combination of winter wet-season precipitation, drying duration, and drying rate (Fig. 6A_4_ and Table 1). On the basis of soil biomarker calibrations, soil temperature closely reflects dry-bulb temperature near surface, particularly in moist soils (forest, woodland, and grassland) that are more thermally conductive than dry soils, and radiative heating on the soil surface is moderated by shading by vegetation and evaporation (*43*, *45*). The modeled dry-bulb surface air temperature (*T*_d_) for the major March to June formation period is ~28°C (Fig. 6A_4_), which is within the error range of our measured *T* (∆_47_) in the lower member of the Niubao Formation.

**Table 1.** Measured stable and clumped isotope results from the Lunpola Basin versus model-predicted carbonate formation periods and paleoelevations under four topographic scenarios for central Tibet during Lutetian and Chattian. Bold italic text indicates most likely scenarios and predicted elevations. Uncertainties are those arising from isotope measurements only. E_2_n^1^, lower member of the Niubao Formation; E_2_n^3^, upper member of the Niubao Formation; δ^18^O_c_, oxygen isotope of carbonates; *T* (Δ_47_), carbonate formation temperature derived from clumped isotopes; δ^18^O_p_, oxygen isotope in paleosurface water; δ^18^O_m_, modeled oxygen isotope in precipitation; *T*_d_, modeled dry-bulb air temperatures during the carbonate formation period; *T_w_*, modeled wet-bulb surface air temperature during the carbonate formation period; *T_w_* msl, modeled wet-bulb surface air temperature at mean sea level during the carbonate formation period.

**Strata** **name**	**Age (Ma)**	**δ^18^O_c_** **(‰)**	***T*(∆_47_)** **(°C)**	**δ^18^O_p_** **(‰)**	**Topography** **scenario**	**δ^18^O_m_** **(‰)**	***T*_d_** **(°C)**	***T*_w_** **(°C)**	***T*_w_ msl** **(°C)**	**Carbonate** **formation** **period**	**Wet bulb** **lapse rate** **(°C/km)**	**Predicted** **elevation (km)**
E_2_n^1^	50–40	−13.2 ± 0.5	23.9 ± 5.9	−11.4 ± 0.5	Central Valley (1.5 km) Gangdese (4.5 km)	−5.1	28	8.3	17.7	***March–June***	3.79	***1.7 + 0.9/−0.8***
					Central watershed (4.0 km)	−6.4	36	8.0	18.6	September	4.6	2.3 ± 0.7
					Central plateau (4.0 km) Gangdese (4.5 km)	−9.0	12	6.7	13.5	March–May	3.29	2.1 ± 1.0
					Central watershed (4.0 km)	−5.7	18	8.0	19.8	September	4.03	2.9 ± 1.0/0–7
E_2_n^3^	37–29	−17.0 ± 1.0	11.9 ± 2.8	−17.4 ± 0.9	Central Valley (2.5 km) Gangdese (5.0 km)	−9.7	24	0.7	15.8	April–June	3.9	3.9 ± 0.4
					Central watershed (5.0 km)	−9.5	22	0.6	16.7	September	2.78	5.8 ± 0.4/−0.8
					Central plateau (4.5 km) Gangdese (5.0 km)	−7.7	14	0.4	18.1	***May–June***	4.29	***4.1 ± 0.4***
					Central watershed (5.0 km)	−11.6	10	1.1	18.8	***September***	4.02	***4.4. ± 0.5/−0.4***

For a Lutetian Tibetan plateau scenario set at 4.0 km and 1120-ppm *p*CO_2_ levels (Fig. 6B), there is again a bimodal precipitation regime, but now most precipitation occurs in the summer (Fig. 6B_1_). However, significant surface evaporation under warm summer air temperatures means soil drying at depth extends from April right through to February (Fig. 6, B_2_ and B_3_). There is no clear period before the summer precipitation peak when nodules could form due to significant soil wetting between February and March (Fig. 6B_3_), with precipitation rising again from April to July (Fig. 6B_1_). The most likely time of nodule formation is September when the modeled *T*_d_ is ~18°C (Fig. 6B_4_), and because this falls outside the range of our measured *T* (∆_47_), we regard this model scenario as not representing past reality.

In a Chattian valley scenario (2.5-km valley floor) with 560-ppm *p*CO_2_ levels, summer precipitation (~20%), like in the Lutetian, is much less than that in the winter (~70%; Fig. 4C_1_). The drying in soil layer 2 precedes that at depth by 2 months and begins in February, immediately precipitation declines (Fig. 4, C_1_ to C_3_). Drying conditions return for the month of September, although the upper soil layers remain dry for most of the summer (Fig. 4C_3_). This regime indicates two possible periods of nodule formation at our sampling depth: in April to May and in September (Fig. 4C_1_). However, the modeled average *T*_d_ during these times are 24° and 22°C, respectively, and so much warmer than the *T*(∆_47_) of 11.9° ± 2.8°C. Again, this disparity suggests that the modeled scenario is unlikely to reflect past conditions.

By contrast, a Chattian plateau (4.5 km) with 560-ppm *p*CO_2_ levels (Fig. 6, D_1_ to D_4_) results in two possible periods of nodule formation: during May to June and again in September, the only uncertainty being the duration of the early summer nodule formation episode given the extended period when evaporation exceeds precipitation despite summer precipitation beginning in June (Fig. 6, D_1_ and D_2_). This would mean that summer meteoric water would not have penetrated far into the soil allowing nodule formation to continue at depth. For this reason, we extend the nodule-forming period into June when the soil was still drying rapidly. We do not extend beyond June because field evidence indicates that the Oligocene soil was more porous and less moisture retentive than the soil simulated in the model. The model soil is a modern standard global average type known as a medium loam and is used because we cannot yet characterize local soils accurately in the water isotope–enabled climate model. The mean *T*_d_s during these intervals are between 11° and 14°C and so similar to the *T* (∆_47_).

### Converting soil temperatures to elevations

Temperatures used in terrestrial thermal lapse rates can be measured in a variety of ways (e.g., dry-bulb or wet-bulb) and averaged over different time intervals (e.g., mean annual, seasonal, or even monthly means) (*33*). These terrestrial lapse rates are location and time dependent, and recent work has demonstrated that the use of thermal lapse rates is far more complex than previously appreciated, so here, we adopt a new approach that overcomes past shortcomings (*33*). Wet-bulb temperature (*T*_w_) is the temperature of an air mass that would result if cooled adiabatically to saturation (no further evaporation and thus cooling can occur). Unlike previous thermal lapse rate approaches that have all used dry-bulb temperatures, those based on *T*_w_ accommodate changes in moisture content as an air parcel traverses a land surface, and in the annual seasonal cycle that controls when soil carbonates form (*33*).

The dry-bulb temperatures (*T*_d_) derived from the clumped isotopes [*T* (∆_47_)] were converted to wet-bulb surface air temperatures (*T*_w_) using modeled humidity data and the Davies-Jones formulation applicable to warm climates (see Materials and Methods for details). We then use geologically consistent terrestrial wet-bulb lapse rates derived from water isotope–enabled climate models for the Eocene and Oligocene that take into account the impact of both moist processes and the period of carbonate formation (see Materials and Methods for details) to reconstruct the height of the Lunpola Basin based on these wet-bulb temperatures. We then apply the same sets of simulations with oxygen-isotope tracking enabled to investigate the relative change in δ^18^O of meteoric water (δ^18^O_m_) that results from changing the elevation of the Central Tibetan Valley from low to high in either the Lutetian or Chattian. This provides a check on our soil carbonate elevation outcomes.

To obtain elevation estimates, we used model-derived mean sea level wet-bulb temperatures (*T*_w_ msl) and terrestrial lapse rates for the same times of the year as the inferred nodule formation. Note that *T*_w_ msl values (Table 1) for the Lutetian are similar to those for the Oligocene, which is seemingly counterintuitive in the context of secular climate change inferred from deep-sea isotopes (*32*). This is because *T*_w_ msl is dependent on evaporative processes, and summer vapor pressure deficit at the closest sea level reference sites in northern India derived from the Climate Leaf Analysis Multivariate Program (CLAMP) (*46*) was higher in the Eocene (the air was drier) than in the Oligocene. The drier Eocene conditions will have induced more evaporative cooling and reduced the *T*_w_ msl for the Eocene to near those of the Oligocene. The difference between the surface *T*_w_ derived from the proxy *T* (Δ_47_) and the model-derived *T*_w_ msl divided by the modeled local *T*_w_ terrestrial lapse rate (again for the specific nodule-forming periods) provides the surface height.

The reconstructed Lutetian elevation based on results for late spring–early summer carbonate formation is 1.7 + 0.9/−0.8 km, and using the much less likely September nodule formation period is 2.3 + 0.7/−0.7 km (Table 1). The uncertainty estimates are based solely on analytical error associated with clumped isotope measurements. This is because uncertainty arising from not knowing precisely the conditions under which the ancient soil carbonates formed, and the uncertainty from water isotope–enabled climate modeling, cannot be fully quantified. Instead, we used four topography sensitivity tests to evaluate whether our elevation results are likely to be realistic. Our low elevation results are almost the same as the 1.5 ± 0.9 km derived by (*16*) for the Early Eocene in the nearby Bangor Basin using conservation of energy principles and moist enthalpy derived from fossil leaf form. If we assume that most soil carbonate precipitation was in March to June as discussed previously, then the clumped isotopes and plant reconstructed elevations are identical within uncertainties, and the isotope dry-bulb temperatures are compatible with those of the model scenarios.

This congruency does not exist for a model set with a Lutetian plateau at 4.0 km where no significant early summer nodule formation is indicated and the September period of formation indicates a surface height of just 2.9 + 1.0/−0.7 km (1σ; Table 1). In this case, both the elevations reconstructed using the wet-bulb lapse rate and surface dry-bulb temperatures are incompatible with the model conditions.

A similar inconsistent outcome is evident for a Chattian valley set at 2.5 km. The *T* (∆_47_) values translate into height estimates of 3.9 ± 0.4 km (1σ) for April to June nodule formation and 5.8 + 0.4/−0.8 km (1σ) for September (Table 1). Notably, the modeled *T*_d_ and clumped isotope dry-bulb temperatures [*T* (Δ_47_)] differ by ~11°C, indicating that the model prescribed elevation conditions are incompatible with clumped isotope–derived dry-bulb temperatures.

When a model is run with a Chattian plateau at 4.5 km, the estimated surface heights are 4.1 ± 0.4 km (1σ) for May to June nodule formation and 4.4 + 0.5/−0.4 km (1σ) for September formation (Table 1). We regard both these times of formation to be equally likely based on soil drying and the precipitation/evaporation regimes (Fig. 6D). These elevations are close to those used in the model, and soil carbonate dry-bulb temperatures are similar, providing confidence in this outcome.

Further validation of modeling scenarios and interpretations comes from a comparison of δ^18^O measurements. The δ^18^O of paleosurface water (δ^18^O_p_) is calculated using the calcite fractionation equation in (*47*) with δ^18^O and *T* (Δ_47_) from carbonates. The δ^18^O_p_ from the lower member of the Niubao Formation has an average value of −11.0 ± 0.5‰ (1σ), which is comparable with the average δ^18^O_p_ value from the negative cluster of the middle member of the Niubao Formation. The upper member of the Niubao Formation has an average δ^18^O_p_ value of −17.4 ± 0.9‰ (1σ), shows a −6.4 ± 0.9‰ (1σ) decrease when compared with the lower member of the Niubao Formation, and matches best with the modeled δ^18^O value of precipitation (δ^18^O_m_) for March to June in the Lutetian valley and to September in the Chattian plateau scenarios, with a −6.5‰ decrease (Table 1 and fig. S8).

The elevations reconstructed from proxies for the Lutetian plateau and Chattian valley scenarios differ markedly from those prescribed in the model (Table 1). In these cases, the model topography is not unduly influencing the outcome of the analysis and suggests that model topography has minimal impact on determining the elevations derived from *T* (Δ_47_). The timing of nodule formation has a much greater impact on the surface height reconstructions, particularly in the case of a hypothetical Chattian valley where there is a difference of almost 2 km in the predicted height depending on whether nodule formation is primarily in late spring–early summer or late summer (Table 1).

It is clear that uncertainties in surface air *T*_w_ and the resultant surface height reconstructions are determined largely by when carbonate precipitation takes place. While not ideal, our somewhat subjective assessment of when nodule formation was likely to have taken place is an improvement over simplistic assumptions that *T* (Δ_47_) represents summertime dry-bulb temperatures, particularly given the bimodal precipitation and drying regimes in the ancient central Tibet (Fig. 6). Lack of knowledge of the exact conditions under which soil carbonates may form makes quantifying uncertainties problematic. Nevertheless, our modeling scenarios quantify bounds on what the carbonate nodules translate to in terms of *T*_w_ values and thus elevation. Our end member topography scenarios also help expose those boundary conditions that are incompatible with proxy data and demonstrate that model Tibetan topography alone does not determine the reconstructed elevations.

## DISCUSSION

### Implications of the revised dating and elevation change scenarios

A robust dating framework is key to documenting any changes in surface height over time. Fang *et al*. (*31*) reported two tuffaceous sandstones from the upper member of the Niubao Formation near the Dayu section, one from below the paleosols dated as 32 Ma and one at the top of the paleosol succession with a 23 Ma age, but they do not provide detailed information for the samples, and the 23-Ma tuffite is the same age as one recorded (*36*) from the overlying Dingqing Formation at Chebuli section. This would suggest either that the lower member of the Dingqing Formation is age-equivalent to parts of the upper member of the Niubao Formation or that the dates in (*31*) are erroneous. The distance between the Chebuli and Dayu sections is merely 15 km in the Lunpola Basin, so the presumption that deposition was diachronous spanning 29 to 23 Ma seems implausible, particularly as evidence for diachronous deposition is lacking in outcrop. We have examined at least 10 targeted dating samples within the upper member of the Niubao Formation at the Dayu section and cannot replicate the 23 Ma result (table S1); the youngest zircon age recovered is 37 Ma (fig. S4E). Moreover, seismic evidence shows that the true thickness of the middle member of the Niubao Formation is ~0.7 km near Dayu area, while the paleomagnetostratigraphic column of (*31*) showed it to be 1.3 km, and so it indicates repeat measurements within the section in outcrop. Last, the upper member of the Niubao Formation contains numerous conglomerates and erosion surfaces as well as the soil-forming episodes that host the carbonate nodules. The unquantified missing time indicated by the erosion surfaces and nondeposition during subaerial soil formation means that the age can only be broadly constrained and also makes the magnetostratigraphic study problematic.

Our lithological and structural analyses of the Dayu section confirm that the palm and fish fossils previously assigned to the younger Dingqing Formation belong to the middle member of the Niubao Formation with an age of no older than ~40 Ma, but no younger than 38 Ma. This is notably older than the 25.5 ± 0.5 Ma assumed in (*17*) and is in agreement with the age assigned to them in (*31*) based on their radiometric dating. This age revision requires a recalculation of the paleoelevation and because the Late Eocene was warmer than the Late Oligocene, as indicated by global proxies (*32*) and in the models, such a reanalysis raises the upper bound of palm survivability from 2.3 to 2.8 km, which is within the surface height bounds based on clumped isotopes presented here. Samples from the upper member of the Niubao Formation have previously yielded high (>4.0 km) Late Eocene paleoelevation estimates based on oxygen and hydrogen isotopes (*25*, *26*), but these estimates need to be re-examined in the context of them now being dated as Early Oligocene.

As well as the effect of dating revisions, the use of clumped isotopes requires knowledge of when in the year the ancient soil carbonates actually formed. In the past, this has been the source of greatest uncertainty (unquantified) and simply assuming that the temperature of formation that reflects mean annual or summer temperatures is clearly not adequate in winter-wet or bimodal precipitation regimes (*44*). By integrating the modeled thermal and moisture regimes to predict when the soil carbonates were most likely to have formed, we reduce such an uncertainty, but even this approach is not yet fully quantifiable.

The bimodal precipitation and surface winds suggesting the mixing of different moisture sources also render previous isotope paleoaltimetry in this region insecure (Fig. 6 and fig. S10). Our new clumped isotope paleoelevations exploit wet-bulb lapse rates derived from modeling, and these show that the Lunpola Basin floor rose from being relatively low (<2 km) during 55 to 38 Ma to more than 4.0 km by 29 Ma. However, the high elevation by the Early Oligocene challenges the leaf wax lipid n-alkanes and palynological evidence from the lower member of the Dingqing Formation, which have been interpreted to record an elevation of ~3 km (*27*, *29*). The difference between our results and those using leaf wax lipid dual isotopes can be explained by the lack of accurate quantification of the apparent fractionation factor in leaf water and paleo-isotopic lapse rates (*17*, *48*). The elevations based on pollen are unlikely to be reliable due to mixing of palynomorphs from different sources at unknown elevations as evidenced by mixture of northwest and southwest surface winds in central Tibet (fig. S9), and the use of an inappropriate dry-bulb free air lapse rate, when such rates are poor at reconstructing past surface heights (*33*).

For the Lutetian valley scenario, our clumped isotope and wet-bulb lapse rate elevations match those of the moist enthalpy results of (*16*) and is below the palm-derived upper limit for the valley floor elevation (*17*) both before and after taking into consideration the revised age determination.

**Fig. 7.**
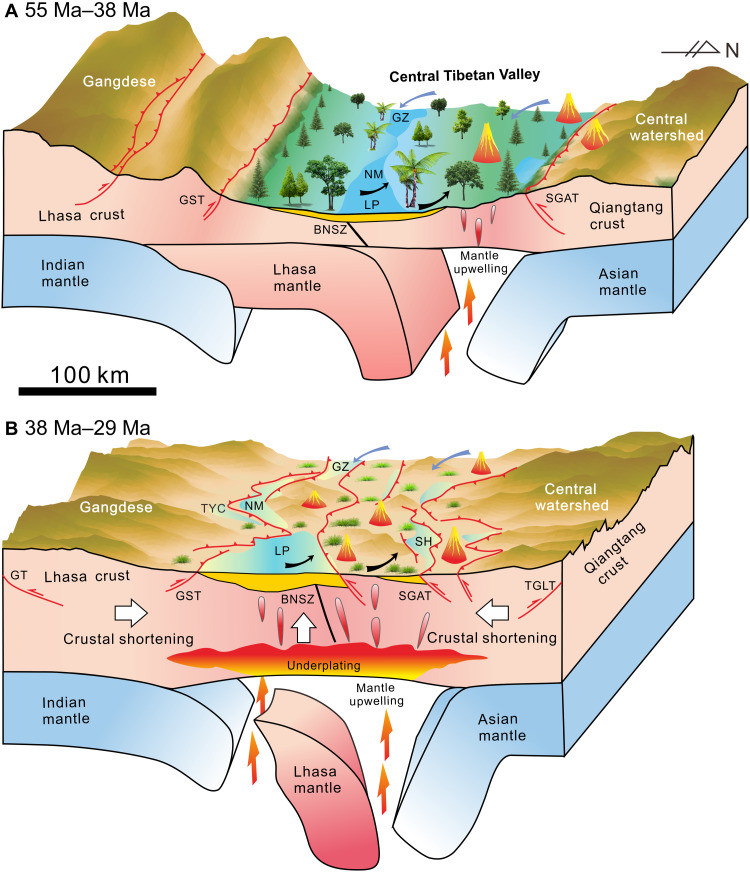
Block models illustrating the topographic evolution of the Central Tibetan Valley and underlying geodynamic processes. The moisture within the valley was sourced by a mix of Westerlies (blue arrows) from the west and monsoonal climate (black arrows) from the southeast. The vertical axis is not to scale. GT, Gandese thrust; TGLT, Tanghula thrust. (**A**) Between ~55 and 38 Ma, the Central Tibetan Valley was a wide lowland with a width of ~200 km sandwiched between the high Gangdese and Central Watershed mountains. A subtropical flora flourished within the valley (*16*). Indentation of the Indian mantle triggered the reactivation of intracontinental subduction of the Lhasa and Songpan-Ganzi terranes, which resulted in E-W Na-rich calc-alkaline volcanics (*51*). (**B**) During 38 to 29 Ma, the ongoing indentation of the Indian mantle lastly caused the detachment of the subducted Lhasa lithospheric mantle. The upwelling of the mantle and immediate basaltic underplating caused lower crustal shortening within the valley, while the heating effect from magmatism in turn promoted upper crustal shortening and deformation. These coupled processes all contributed to rapid surface uplift within the Central Tibetan Valley to raise the surface to over 4.0 km. Forest vegetation diminished and herbaceous vegetation, including grasses, thrived (*56*).

Combined with paleoelevation data from the Gangdese and Central Watershed mountains, this suggests that, during Early to Middle Eocene time, the topography of what now is the central Tibetan Plateau presented as Gangdese and Central Watershed highlands sandwiching a Central Tibetan Valley [Fig. 7A; (*8*, *14*–*16*)]. The valley floor rose rapidly by ~2.4 to 2.7 km during the Late Eocene to Early Oligocene at a rate of 0.24 to 0.27 mm/year, and central Tibet likely reached its near-present elevation in the Late Oligocene (Fig. 7B) and thus just before the Neogene.

### Valley demise mechanisms

Alternative explanations involving mantle upwelling and crustal shortening (*2*, *6*, *18*) can be advanced to explain the 2.4- to2.7-km, or more, surface uplift of the Central Tibetan Valley during the Paleogene.

Mantle upwelling predicts surface elevation could rise more than 2 km through removal of mantle lithosphere and further melting of lower crust (*2*, *6*). Seismic evidence suggests an upper-mantle remnant from earlier lithospheric foundering in central Tibet (*49*) and implies that mantle upwelling could have played an important role in the uplift of the Central Tibetan Valley. The basaltic underplating coupled with mantle upwelling through melting of lower crust has been shown to play a comparable role in 15-km crustal thickening of northern Tibet (*50*), while the east-west trending Na-rich calc-alkaline lavas produced during 38 to 27 Ma in the southern Qiangtang terrane are indicative of syn-collisional mantle melting processes (*51*).

Crustal shortening follows the rule of isostasy compensation, so the >2-km surface rise in the Lunpola Basin during 40 to 30 Ma requires at least ~15-km crustal thickening. The Central Tibetan Valley has been deformed strongly by the N-dipping Shiquanhe-Gaize-Amdo thrust and S-dipping Gaize-Siling Co thrust, and structural restoration suggests a minimum of ~28% shortening in the Middle Eocene to Late Oligocene (43 to 26 Ma) (*14*, *23*, *52*). Paleomagnetic evidence from central Tibet also suggests that more than 1000 km of intracontinental convergence took place during the Eocene to Early Oligocene (*53*). Therefore, regional uplift of the Central Tibetan Valley during the Paleogene could result from substantial upper crustal shortening within the region during this period (*14*, *23*, *52*, *53*).

The synchronous calc-alkaline magmatism and crustal shortening in the Central Tibetan Valley (*14*, *23*, *52*, *53*), and their interaction with mantle melting, enhanced thermal weakening specifically in the southern Qiangtang terrane (*51*) and lead us to propose that mantle upwelling and crustal shortening together contributed to the >2-km Late Paleogene rise of the Central Tibetan Valley.

Our concept of the processes driving the demise of the Central Tibetan Valley is summarized as follows and in Fig. 7. Between ~55 and 38 Ma, the Central Tibetan Valley was a broad lowland <2.0 km above mean sea level and ~200-km wide. Indentation of the Indian mantle reactivated the intracontinental subduction of the Lhasa and Songpan-Ganzi terranes underneath the Qiangtang terrane, which resulted in East to West Na-rich calc-alkaline volcanics in the southern Qiangtang terrane (Fig. 7A). Between 38 and 29 Ma, the ongoing indentation of the Indian mantle lastly caused detachment of the subducted Lhasa mantle. The upwelling of mantle and rapid basaltic underplating caused magmatism and crustal shortening within the thermally weakened lower crust. The magmatic heating in turn promoted upper crustal shortening within the valley. The interactions between mantle upwelling and crustal shortening resulted in the rapid rise of the Central Tibetan Valley floor to more than 4.0 km before the Neogene (Fig. 7B).

### Asian climate and biodiversity implications

The present dry-bulb mean annual air temperature (MAAT) of the Lunpola Basin is ~−1°C, and the annual precipitation ranges from 300 to 500 mm with summer rainfall dominating the precipitation regime. The bimodal precipitation pattern evident in the Lutetian valley and Chattian plateau scenarios (Fig. 6) indicates a complex hydroclimate within the central Tibetan region during the Paleogene (Fig. 7), which challenges the idea of a South Asian monsoonal regime penetrating into Tibet as at present, and suggests multiple moisture sources. To further refine future isotope paleoaltimetry, it will be necessary to use isotope-enabled GCMs to track moisture trajectories (*54*). While these models are not perfect, they offer a more objective way forward than the “educated guesses” often used in many isotope studies at present (*55*).

Our modeling results indicate that the dry-bulb MAAT of the central Tibet during the Lutetian was ~20°C and thus consistent with CLAMP results from the Middle Eocene Jianglang flora in the nearby Bangor Basin (*16*), while the MAAT during Oligocene time, for which there are as yet no leaf–based proxy temperature data, was likely to have been close to 0°C (fig. S10), which is comparable with the present MAAT in the Lunpola Basin. The decrease in MAAT from the Middle Eocene through the Oligocene implies a significant drop in winter temperatures and an increase in the frequency and duration of freezing conditions that would have exerted a radical change in biodiversity within central Tibet (*16*, *17*, *27*, *28*, *30*). During the Middle Eocene, the Central Tibetan Valley system hosted a subtropical ecosystem as indicated by CLAMP analysis of leaf form, and the recovered plant assemblages include palms such as *Sabalites* (*17*) and taxa-like *Lagokarpos* (*16*). The biota also includes climbing perches whose physiology is intolerant of lake surface freezing (*30*). However, by the Early Oligocene, central Tibet had transformed into a cold high plateau (*27*, *56*) and the role of central Tibet as a biodiversity exchange route and nursery for lowland taxa withered (*56*). We note that there is an apparent gap in the plant fossil record during deposition of the upper member of the Niubao Formation and before the Late Oligocene warming. This may reflect sparse vegetation or a comparative lack of paleobotanical exploration. As Tibetan temperatures recovered in the Late Oligocene, the region became repopulated by plants adapted to a cool temperate climate as evidenced by pollen records from the lower member of the Dingqing Formation, which points to the presence of a mostly deciduous broadleaved cool temperate woodland (*27*, *56*).

## MATERIALS AND METHODS

### General approach

Within a rigorous chronostratigraphic framework constructed from detailed field mapping, stratigraphic and sedimentological analysis at four sections (Figs. 1 and 3 and figs. S1 to S3 and S5), seismic data (Fig. 2), and radiometric dating (Fig. 4 and fig. S4), we use water isotope–enabled climate models to predict when in the year soil carbonates were likely to form under end-member topographic scenarios encompassing a low Central Tibetan Valley or a high plateau. Each end-member topographic simulation creates a different thermal and hydrological regime at the land surface and within the soil (Fig. 6). These different topographies modify the local and regional atmospheric circulation, land surface–atmosphere interactions, and in turn temperature and precipitation patterns at the surface and soil subsurface. We then determine near-surface dry-bulb air temperatures (*T*_d_) from carbonate-clumped isotopes (*38*) and then wet-bulb surface air temperatures (*T*_w_) using model-derived atmospheric conditions (*33*). Last, we derive surface heights using local wet-bulb thermal lapse rates at Earth’s surface for the time(s) in the year, when carbonate was likely to form calculated from geologic stage–specific climatic simulations (Fig. 6 and figs. S7 to S10).

### Seismic data interpretation

The analyses of the structural style and the calibration of sedimentary strata within the Dayu section are primarily based on seismic data provided by Sinopec Southern Branch (China) (*56*). We selected two-dimensional (2D) seismic profiles that cover most structures and strata in the central region of the Lunpola Basin (Fig. 2 and fig. S11). The original seismic data, pre-stack time-migration SEG-Y (Society of Exploration Geophysicists) files, were integrated into and interpreted by the commercial software, King-dom (HIS). This 2D stack-migration seismic profile (*57*) is converted from time to depth with an average speed of ~3000 m/s to facilitate intuitive interpretation.

The seismic reflection boundaries between the sedimentary cover and basement, and between Cenozoic and Mesozoic strata, are clear. Cenozoic strata are present throughout the entire basin and are recognized as the lower, middle, and upper members of the Niubao Formation and the lower, middle, and upper members of the Dingqing Formation (from bottom to top) (Fig. 2 and fig. S11). In our work, the calibration of each seismic stratigraphic layer was constrained precisely by synthetic seismograms and substantiated by logging and borehole records from three wells [W1 to W3, (*57*)], followed by lateral comparison and tracking. The depiction of the faults was strictly based on the offset of seismic layers in the sedimentary cover, surface outcrop fault spread and was inferred near the basement. The geological map of the Lunpola Basin is used to calibrate the surface of the seismic profile, so that the surface structure along the survey line is evident (Fig. 1B), and the location and occurrence of the main faults can be controlled.

### Zircon U-Pb geochronology

Zircon grains were prepared using standard separation techniques, mounted in epoxy, and then polished to expose grain interiors. The polished zircons were examined in cathodoluminescence mode at the State Key Laboratory of Tibetan Plateau Earth System, Environment and Resources (TPESER), Institute of Tibetan Plateau Research, Chinese Academy of Sciences (ITPCAS) using a JEOL JXA-8100 Super Probe scanning electron microscope for characterizing potential complexities. U-Pb dating was performed in situ with a New Wave UP 193FX Excimer laser (New Wave Instrument, USA) coupled to an Agilent 7500a inductively coupled plasma mass spectrometer [laser ablation inductively coupled plasma mass spectrometry (LA-ICP-MS)] installed at ITPCAS. Zircon ablation used a 35-μm spot diameter, 8-Hz repetition, and ~5 to 8 J/cm^2^ energy. Standard zircons used for calibration were Plešovice (337 Ma) and 91500 (1064 Ma) (*58*). Offline isotope ratios and trace element concentrations were calculated using GLITTER 4.0 (*59*). The U-Pb isotope results of volcanic samples are placed on the Wetherill-type Concordia diagram with 1σ, and the weighted Concordia ages are calculated with 2σ presented in Fig. 4. The U-Pb isotope results of sandstones are shown with probability density, and the youngest zircon ages in fig. S4. All U-Pb data are provided in table S1.

### Carbonate stable isotope analysis

The paleosol nodules and lacustrine carbonate samples collected for stable and clumped isotope analysis were all from the Niubao Formation. All paleosol nodules were sampled from a depth of >40 cm below the upper horizon of the paleosol to reduce the uncertainties arising from postformation alteration and CO_2_ exchange (*60*). The collected samples were powdered using a microdrill with a tungsten bur at low speed to avoid overheating and potential C-O redistribution.

The carbon and oxygen isotopic analyses were conducted on an IsoPrime100 gas source stable isotope ratio MS (IRMS) equipped with a MultiPrep system at the Speleothem Laboratory, Institute of Earth Environment, Chinese Academy of Science. The stable isotope results are normalized to NBS-19 in conventional delta notation (δ^13^C_c_ and δ^18^O_c_) and reported relative to the Vienna Peedee Belemnite (*61*). The uncertainties (2σ) are better than 0.08 and 0.06‰ for carbon and oxygen isotopes, respectively. All stable isotope data are provided in table S2.

### Carbonate clumped isotope analysis

Clumped isotope paleothermometry is based on the principle that the formation temperature of carbonate is proportional to the relative enrichment of isotopologue ^13^C^18^O^16^O (i.e., Δ_47_) in carbonates (*62*). Δ_47_ is independent of the isotopic compositions of water from which the carbonate grew, thus providing a temperature-dependent approach to determine the paleoelevation (*62*).

Twelve paleosol carbonates checked for diagenesis (see details in the Supplementary Materials) from the Niubao Formation were chosen for clumped isotope analysis. Six nodules were collected from the interbedded paleosols of the lower member of the Niubao Formation (2015TL28, 2015TL108, 2021TL01, 2021TL02, 2021TL03, and 2021TL04; fig. S2), four nodules were collected from the upper member of the Niubao Formation (2015TL33, 2015TL86, 2015TL119, and 2017TX27; fig. S2), and two marls were collected from the middle member of the Niubao Formation (2015TL74 and 2015TL76; fig. S2).

Clumped isotope analyses were conducted in the Department of the Geophysical Sciences at the University of Chicago and TPESER at ITPCAS. At the University of Chicago, the powdered carbonate samples were digested in ~9 ml of anhydrous phosphoric acid (density, 1.92 to 1.94 g/ml) at 26°C overnight. All the acid-released CO_2_ samples were extracted through a liquid nitrogen trap and then purified on a vacuum line to remove potential contaminants including water vapor, hydrocarbons, and halocarbons by passing through three cryogenically traps in sequence, namely, a water trap (glass tube immersed in liquid nitrogen-ethanol slurry at −80°C), a packed static trap (glass trap filled with PoraPak Q hold at −10°C), and another water trap (glass tube immersed in liquid nitrogen-ethanol slurry at −80°C). The purified CO_2_ samples were measured on a Finnigan MAT 253 gas source IRMS configured to simultaneously collect mass/charge ratio 44 to 49 ion beams to obtain the raw Δ_47_ data. All acquired raw Δ_47_ data were corrected for nonlinearity and pressure baseline background effects of MS through laboratory empirical calibration and then standardized to an absolute reference frame (*63*).

The methodology in the ITPCAS is similar to that of the University of Chicago, except that the powdered carbonate samples were reacted with 1 to 2 ml of anhydrous phosphoric acid (ρ = ~1.93 g/cm^3^) at 90°C for 15 min and that the measurement of Δ_47_ is conducted on a 235 Plus IRMS. We further construct a standard transfer function to standardize our samples and the effect of acid digestion at 90°C (*64*).

The precision of individual analysis was better than 0.013‰ (1σ) and the analytical temperature uncertainties of temperatures were propagated through the calibration equation in (*38*). All the data are provided in tables S3 and S5.

### Model simulations

General circulation models (GCMs) have long been used to forecast weather and climate conditions with skill; however, their application to geological time periods is problematic due to uncertainties in model boundary conditions, some of which are well constrained (solar luminosity) and some are not (precise atmospheric composition).

The topographic height and structure of Tibet (a boundary condition supplied to the model) is the largest source of uncertainty in regard to atmospheric circulation and land surface processes in our study. This is because of the different responses of the atmosphere (including mass, momentum, and energy fluxes; regional temperature; and hydrological response) to a plateau versus a twin mountain ranges bounding a central valley system. By running climate models with different topographic boundary conditions, we are able to better constrain this source of uncertainty for predicted local dry- and wet-bulb lapse rates and provide uncertainty bounds for our analyses.

Four topographic scenarios were devised based on existing end-member topographic reconstructions reviewed in (*56*). The first is that the core of Tibet was already high (≥4.5 km) by 40 Ma (Eocene) and that further uplift subsequently occurred both to the north and to the south, an idea first proposed by Wang *et al*. (*8*). Our second configuration, a complex dual mountain system with a central low-lying valley, was proposed by Ding *et al*. (*7*) and Su et al. (*17*) and theorized to have existed and spanned the Eocene–Early Miocene.

We used a fully coupled atmosphere-ocean GCM (HadCM3BL-M2.1aD) (*65*) with a spatial resolution of 2.5 × 3.75 latitude by longitude and comprising 19 vertical levels in the atmosphere and 20 vertical levels in the ocean. HadCM3BL-M2.1aD has shown skill in reproducing both modern Quaternary and deep-time climates compared to observations and has seen continual development to improve simulation skill since its inception [see (*65*) for details]. Carbon dioxide concentrations were chosen based on proxy-CO_2_ reconstructions from (*66*) and, in general, bracket both the high and low end of the reconstructions (95% confidence level) so that the full impact of variable *p*CO_2_ can be assessed.

Four model simulations with Lutetian and Chattian stage–specific boundary conditions [topography, bathymetry, solar luminosity, and continental ice; see (*67*) for further details] at 2× preindustrial CO_2_ and 4× preindustrial CO_2_ using a base state Getech paleogeography were performed. Although the date has been revised to the Rupelian, we use a Chattian paleogeography due to both time periods being very similar in terms of their respective configuration (ice sheets, topography, and bathymetry) (*67*, *68*). The Chattian represents the upper bound of the Rupelian scenario. Oligocene modeled temperatures were almost identical (difference in MAAT for Rupelian and Chattian conditions was just 1.2°C) for a plateau scenario and because we had already explored a range of Oligocene topographies and their effects (*17*). Each simulation was run for 10,422 model years to ensure full climate equilibrium and no trend in temperatures in the atmosphere and deep ocean, as well as energy balance at the top of the atmosphere (*1*). Initialized from these simulations, four idealized topographies were constructed (fig. S7) and implemented in an isotope-enabled version of HadCM3BL-M2.1aD (*69*) that has been shown to successfully simulate both modern and past Eocene δ^18^O distribution of seawater and carbonate proxy data.

Climate simulations for Lutetian and Chattian Tibet are carried out in four scenarios (summarized in table S4) to determine the consequence of the surface air temperature and δ^18^O change in precipitation (δ^18^O_m_), so as to verify the paleoelevation results. Scenario 1 sees a Lutetian (~44 Ma) Tibet with a central low valley at 1.5 km surrounded by the 4.5-km Gangdese mountains to the south and the 4.0-km Central Watershed mountains to the north (fig. S7A). Scenario 2 is for a Lutetian (~44 Ma) Tibet, where the central part is elevated to 4.0 km (fig. S7B), the Gangdese mountains to 4.5 km to the South, and the Central Watershed mountains to 4.0 km to the North; that is to say, the same as in scenario 1. Both scenarios 1 and 2 were run at 1120-ppm *p*CO_2_. Scenario 3 (fig. S7C) envisages a Chattian (~25 Ma) Tibet with a low central valley at 2.5 km, between 5-km high Gangdese and Central Watershed mountains to the south and north, respectively. Last, scenario 4 (fig. S7D) is a Chattian (~25 Ma) Tibet with a high elevated central landform at 4.5 km, bounded by 5-km high Gangdese and Central Watershed mountains to the south and north, respectively. Both scenarios 3 and 4 were run at 560-ppm *p*CO_2_. Each water isotope–enabled climate model scenario was run for an additional 60 model years with the last 30 years used to generate the mean isotopic signal.

It is not yet possible to reconstruct ancient soil types with any accuracy on a global scale, so the soil characteristics used in our model reflect those of a midlatitude loam with perhaps higher organic content than the ancient low latitude and coarser Tibetan soils. The use of a loam involves higher water retention capacity than was the case for our Paleogene soils, but by using criteria other than simple soil moisture to define when nodule formation was most likely to have taken place at depth within the soil, we argue that we better define the relationship between *T* (Δ_47_) and seasonal air temperatures than in previous studies (*41*–*42*).

To evaluate the relationship between topographic rise, temperature decrease, and δ^18^O_p_ changes in central Tibet, we further examined four isotope fractionation and transport scenarios using an isotope-enabled GCM under the four scenarios above. The isotope-enabled HadCM3L GCM has shown prior skill in both reproducing modern and Early Eocene δ^18^O distributions from modern observations and geologic proxies (*69*). This approach also helps exclude scenarios incompatible with proxy data.

### Wet-bulb GCM-derived lapse rates

A recent assessment of thermal lapse rate paleoaltimetry techniques has shown that conventional dry-bulb temperatures (*T*_d_), which ignore moist processes, lead to large uncertainties in height estimates, whereas wet-bulb temperatures (*T*_w_) and lapse rates, irrespective of season, show considerable skill at reproducing prescribed landscapes (*33*). Moreover, unlike many previous attempts at deriving surface height through thermal lapse rates, we do not use temperature changes in a nonconvecting column of free air (an environmental lapse rate), but rather the change in temperature with changing elevation near Earth’s surface (a terrestrial lapse rate) where proxy records form. Here, we apply wet-bulb–derived terrestrial lapse rates to ascertain height estimates for central Tibet.

Wet-bulb temperatures were calculated through the Davies-Jones formulation that is more accurate for warm climate of the past using climate model derived *T*_d_, relative humidity and surface pressure (*70*; equation 38). Full derivation of the wet-bulb formulation can be found in (*71*; equation 3.8). To obtain the paleoelevations from the difference in the *T*_w_ at sea level (*T*_w_ msl) and *T*_w_ values at our research site, we used water isotope–enabled climate model–derived Lutetian and Oligocene local terrestrial lapse rates for central Tibet appropriate for the times of the year when nodules formed (to a temporal resolution of 1 month). Because orography is only known approximately and is what we are trying to better quantify, we use a sensitivity study approach where we explore end-member conditions. These provide bounds for surface height estimates and eliminate unrealistic reconstructions.

## Supplementary Materials

### This PDF file includes:

Supplementary Materials Figs. S1 to S11 Legends for tables S1 to S5 References

## References

[1] A. Farnsworth, D. J. Lunt, S. A. Robinson, P. J. Valdes, W. H. G. Roberts, P. D. Clift, P. Markwick, T. Su, N. Wrobel, F. Bragg, S. Kelland, R. D. Pancost, Past East Asian monsoon evolution controlled by paleogeography, not CO_2_. Sci. Adv. 5, eaax1697 (2019). 31692956 10.1126/sciadv.aax1697 PMC6821471

[2] P. Molnar, P. England, J. Martinod, Mantle dynamics, uplift of the Tibetan Plateau, and the Indian monsoon. Rev. Geophys. 31, 357–396 (1993).

[3] S.-F. Li, P. J. Valdes, A. Farnsworth, T. Davies-Barnard, T. Su, D. J. Lunt, R. A. Spicer, J. Liu, W.-Y.-D. Deng, J. Huang, H. Tang, A. Ridgwell, L.-L. Chen, Z.-K. Zhou, Orographic evolution of northern Tibet shaped vegetation and plant diversity in eastern Asia. Sci. Adv. 7, eabc7741 (2021). 33571113 10.1126/sciadv.abc7741 PMC7840128

[4] P. Molnar, W. R. Boos, D. S. Battisti, Orographic controls on climate and paleoclimate of Asia: Thermal and mechanical roles for the Tibetan Plateau. Annu. Rev. Earth Planet. Sci. 38, 77–102 (2010).

[5] T. M. Harrison, P. Copeland, W. S. F. Kidd, A. Yin, Raising Tibet. Science 255, 1663–1670 (1992). 17749419 10.1126/science.255.5052.1663

[6] P. England, G. Houseman, Extension during continental convergence, with application to the Tibetan Plateau. J. Geophys. Res. 94, 17561–17579 (1989).

[7] L. Ding, Q. Xu, Y.-H. Yue, H.-Q. Wang, F.-L. Cai, S. Li, The Andean-type Gangdese Mountains: Paleoelevation record from the Paleocene-Eocene Linzhou Basin. Earth Planet. Sci. Lett. 392, 250–264 (2014).

[8] C.-S. Wang, X.-X. Zhao, Z.-F. Liu, P. C. Lippert, S. A. Graham, R. S. Coe, H.-S. Yi, L.-D. Zhu, S. Liu, Y.-L. Li, Constraints on the early uplift history of the Tibetan Plateau. Proc. Natl. Acad. Sci. U.S.A. 105, 4987–4992 (2008). 18362353 10.1073/pnas.0703595105 PMC2278176

[9] A. Mulch, C. P. Chamberlain, The rise and growth of Tibet. Nature 439, 670–671 (2006). 16467826 10.1038/439670a

[10] L. H. Royden, B. C. Burchfiel, R. D. van der Hilst, The geological evolution of the Tibetan plateau. Science 321, 1054–1058 (2008). 18719275 10.1126/science.1155371

[11] P. Tapponnier, Z.-Q. Xu, F. Roger, B. Meyer, N. Arnaud, G. Wittlinger, J.-S. Yang, Oblique stepwise rise and growth of the Tibet plateau. Science 294, 1671–1677 (2001). 11721044 10.1126/science.105978

[12] P. England, G. Houseman, Finite strain calculations of continental deformation 2. Comparison with the India-Asia collision zone. J. Geophys. Res. 91, 3664–3676 (1986).

[13] C.-S. Wang, J.-G. Dai, X.-X. Zhao, Y.-L. Li, S. A. Graham, D.-F. He, B. Ran, J. Meng, Outward-growth of the Tibetan Plateau during the Cenozoic: A review. Tectonics 621, 1–43 (2014).

[14] P. Kapp, P. G. DeCelles, G. E. Gehrels, M. Heizler, L. Ding, Geological records of the Lhasa-Qiangtang and Indo-Asian collisions in the Nima area of central Tibet. Geol. Soc. Am. Bull. 119, 917–933 (2007).

[15] Z.-Y. Xiong, L. Ding, R. A. Spicer, A. Farnsworth, X. Wang, P. J. Valdes, T. Su, Q.-H. Zhang, L.-Y. Zhang, F.-L. Cai, H.-Q. Wang, Z.-Y. Li, P.-P. Song, X.-D. Guo, Y.-H. Yue, The early Eocene rise of the Gonjo Basin, SE Tibet: From low desert to high forest. Earth Planet. Sci. Lett. 543, 116312 (2020).

[16] T. Su, R. A. Spicer, F.-X. Wu, A. Farnsworth, J. Huang, C. Del Rio, T. Deng, L. Ding, W.-Y.-D. Deng, Y.-J. Huang, A. Hughes, L.-B. Jia, J.-H. Jin, S.-F. Li, S.-Q. Liang, J. Liu, X.-Y. Liu, S. Sherlock, T. Spicer, G. Srivastava, H. Tang, P. Valdes, T.-X. Wang, M. Widdowson, M.-X. Wu, Y.-W. Xing, C.-L. Xu, J. Yang, C. Zhang, S.-T. Zhang, X.-W. Zhang, F. Zhao, Z.-K. Zhou, A Middle Eocene lowland humid subtropical "Shangri-La" ecosystem in central Tibet. Proc. Natl. Acad. Sci. U.S.A. 117, 32989–32995 (2020). 33288692 10.1073/pnas.2012647117 PMC7777077

[17] T. Su, A. Farnsworth, R. A. Spicer, J. Huang, F.-X. Wu, J. Liu, S.-F. Li, Y.-W. Xing, Y.-J. Huang, W.-Y.-D. Deng, H. Tang, C.-L. Xu, F. Zhao, G. Srivastava, P. J. Valdes, T. Deng, Z.-K. Zhou, No high Tibetan Plateau until the Neogene. Sci. Adv. 5, eaav2189 (2019). 30854430 10.1126/sciadv.aav2189 PMC6402856

[18] A. Yin, T. M. Harrison, Geologic evolution of the Himalayan-Tibetan orogen. Annu. Rev. Earth Planet. Sci. 28, 211–280 (2000).

[19] P. Kapp, P. G. DeCelles, Mesozoic-Cenozoic geological evolution of the Himalayan-Tibetan orogen and working tectonic hypotheses. Am. J. Sci. 319, 159–254 (2019).

[20] C. Chengfa, C. Nansheng, M. P. Coward, D. Wanming, J. F. Dewey, A. Gansser, N. B. W. Harris, J. Chengwei, W. S. F. Kidd, M. R. Leeder, L. Huan, L. Jinlu, L. Chengjie, M. Houjun, P. Molnar, P. Yun, P. Yusheng, J. A. Pearce, R. M. Shackleton, A. B. Smith, S. Yiyin, M. Ward, D. R. Watts, X. Juntao, X. Ronghua, Y. Jixiang, Z. Yuquan, Preliminary conclusions of the Royal Society and Academia Sinica 1985 Geotraverse of Tibet. Nature 323, 501–507 (1986).

[21] M. Taylor, A. Yin, F. J. Ryerson, P. Kapp, L. Ding, Conjugate strike-slip faulting along the Bangong-Nujiang suture zone accommodates coeval east-west extension and north-south shortening in the interior of the Tibetan Plateau. Tectonics 22, 1044 (2003).

[22] A. Yin, M. H. Taylor, Mechanics of V-shaped conjugate strike-slip faults and the corresponding continuum mode of continental deformation. Geol. Soc. Am. Bull. 123, 1798–1821 (2011).

[23] P. Kapp, M. A. Murphy, A. Yin, T. M. Harrison, L. Ding, J. Guo, Mesozoic and Cenozoic tectonic evolution of the Shiquanhe area of western Tibet. Tectonics 22, 1029 (2003).

[24] Y. Wei, K. Zhang, C. N. Garzione, Y. Xu, B. Song, J. Ji, Low palaeoelevation of the northern Lhasa terrane during late Eocene: Fossil foraminifera and stable isotope evidence from the Gerze Basin. Sci. Rep. 6, 27508 (2016). 27272610 10.1038/srep27508 PMC4897749

[25] D. B. Rowley, B. S. Currie, Palaeo-altimetry of the late Eocene to Miocene Lunpola basin, central Tibet. Nature 439, 677–681 (2006). 16467830 10.1038/nature04506

[26] P. J. Polissar, K. H. Freeman, D. B. Rowley, F. A. McInerney, B. S. Currie, Paleoaltimetry of the Tibetan Plateau from *D/H* ratios of lipid biomarkers. Earth Planet. Sci. Lett. 287, 64–76 (2009).

[27] J. Sun, Q. Xu, W. Liu, Z. Zhang, L. Xue, P. Zhao, Palynological evidence for the latest Oligocene–early Miocene paleoelevation estimate in the Lunpola Basin, central Tibet. Palaeogeogr. Palaeoeclimatol. Palaeoecol. 399, 21–30 (2014).

[28] T. Deng, S. Wang, G. Xie, Q. Li, S. Hou, B. Sun, A mammalian fossil from the Dingqing Formation in the Lunpola Basin, northern Tibet, and its relevance to age and paleo-altimetry. Chin. Sci. Bull. 57, 261–269 (2011).

[29] G. Jia, Y. Bai, Y. Ma, J. Sun, P. Peng, Paleoelevation of Tibetan Lunpola basin in the Oligocene-Miocene transition estimated from leaf wax lipid dual isotopes. Global Planet. Change 126, 14–22 (2015).

[30] F. Wu, D. Miao, M.-m. Chang, G. Shi, N. Wang, Fossil climbing perch and associated plant megafossils indicate a warm and wet central Tibet during the late Oligocene. Sci. Rep. 7, 878 (2017). 28408764 10.1038/s41598-017-00928-9 PMC5429824

[31] X. Fang, G. Dupont-Nivet, C. Wang, C. Song, Q. Meng, W. Zhang, J. Nie, T. Zhang, Z. Mao, Y. Chen, Revised chronology of central Tibet uplift (Lunpola Basin). Sci. Adv. 6, eaba7298 (2020). 33298435 10.1126/sciadv.aba7298 PMC7725450

[32] T. Westerhold, N. Marwan, A. J. Drury, D. Liebrand, C. Agnini, E. Anagnostou, J. S. K. Barnet, S. M. Bohaty, D. De Vleeschouwer, F. Florindo, T. Frederichs, D. A. Hodell, A. E. Holbourn, D. Kroon, V. Lauretano, K. Littler, L. J. Lourens, M. Lyle, H. Pälike, U. Röhl, J. Tian, R. H. Wilkens, P. A. Wilson, J. C. Zachos, An astronomically dated record of Earth’s climate and its predictability over the last 66 million years. Science 369, 1383–1387 (2020). 32913105 10.1126/science.aba6853

[33] A. Farnsworth, P. J. Valdes, R. A. Spicer, L. Ding, C. Witkowski, V. Lauretano, T. Su, S. Li, S. Li, Z. Zhou, Paleoclimate model-derived thermal lapse rates: Towards increasing precision in paleoaltimetry studies. Earth Planet. Sci. Lett. 564, 116903 (2021).

[34] A. K. Laskowski, D. A. Orme, F.-L. Cai, L. Ding, The Ancestral Lhasa River: A Late Cretaceous trans-arc river that drained the proto–Tibetan Plateau. Geology 47, 1029–1033 (2019).

[35] H. He, J. Sun, Q. Li, R. Zhu, New age determination of the Cenozoic Lunpola basin, central Tibet. Geol. Mag. 149, 141–145 (2012).

[36] P. G. DeCelles, J. Quade, P. Kapp, M. Fan, D. L. Dettman, L. Ding, High and dry in central Tibet during the Late Oligocene. Earth Planet. Sci. Lett. 253, 389–401 (2007).

[37] C. N. Garzione, G. D. Hoke, J. C. Libarkin, S. Withers, B. MacFadden, J. Eiler, P. Ghosh, A. Mulch, Rise of the Andes. Science 320, 1304–1307 (2008). 18535236 10.1126/science.1148615

[38] J. R. Kelson, K. W. Huntington, A. J. Schauer, C. Saenger, A. R. Lechler, Toward a universal carbonate clumped isotope calibration: Diverse synthesis and preparatory methods suggest a single temperature relationship. Geochim. Cosmochim. Acta 197, 104–131 (2017).

[39] H. Li, X. Liu, A. Arnold, B. Elliott, R. Flores, A. M. Kelley, A. Tripati, Mass 47 clumped isotope signatures in modern lacustrine authigenic carbonates in Western China and other regions and implications for paleotemperature and paleoelevation reconstructions. Earth Planet. Sci. Lett. 562, 116840 (2021).

[40] M. R. Talbot, A review of the palaeohydrological interpretation of carbon and oxygen isotopic ratios in primary lacustrine carbonates. Chem. Geol. 80, 261–279 (1990).

[41] J. R. Kelson, K. W. Huntington, D. O. Breecker, L. K. Burgener, T. M. Gallagher, G. D. Hoke, S. V. Petersen, A proxy for all seasons? A synthesis of clumped isotope data from Holocene soil carbonates. Quat. Sci. Rev. 234, 106259 (2020).

[42] J. Quade, J. Eiler, M. Daëron, H. Achyuthan, The clumped isotope geothermometer in soil and paleosol carbonate. Geochim. Cosmochim. Acta 105, 92–107 (2013).

[43] M. C. Ringham, G. D. Hoke, K. W. Huntington, J. N. Aranibar, Influence of vegetation type and site-to-site variability on soil carbonate clumped isotope records, Andean piedmont of Central Argentina (32–34°S). Earth Planet. Sci. Lett. 440, 1–11 (2016).

[44] D. O. Breecker, Z. D. Sharp, L. D. McFadden, Seasonal bias in the formation and stable isotopic composition of pedogenic carbonate in modern soils from central New Mexico, USA. Geol. Soc. Am. Bull. 121, 630–640 (2009).

[45] H. Lu, W. Liu, H. Yang, H. Wang, Z. Liu, Q. Leng, Y. Sun, W. Zhou, Z. An, 800-kyr land temperature variations modulated by vegetation changes on Chinese Loess Plateau. Nat. Commun. 10, 1958 (2019). 31036861 10.1038/s41467-019-09978-1 PMC6488643

[46] H. Bhatia, G. Srivastava, R. A. Spicer, A. Farnsworth, T. E. V. Spicer, R. C. Mehrotra, K. N. Paudayal, P. Valdes, Leaf physiognomy records the Miocene intensification of the South Asia Monsoon. Global Planet. Change 196, 103365 (2021).

[47] S.-T. Kim, J. R. O’Neil, Equilibrium and nonequilibrium oxygen isotope effects in synthetic carbonates. Geochim. Cosmochim. Acta 61, 3461–3475 (1997).

[48] F. A. Smith, K. H. Freeman, Influence of physiology and climate on δD of leaf wax *n*-alkanes from C_3_ and C_4_ grasses. Geochim. Cosmochim. Acta 70, 1172–1187 (2006).

[49] M. Chen, F. Niu, J. Tromp, A. Lenardic, C.-T. A. Lee, W. Cao, J. Ribeiro, Lithospheric foundering and underthrusting imaged beneath Tibet. Nat. Commun. 8, 15659 (2017). 28585571 10.1038/ncomms15659 PMC5467168

[50] J.-L. Chen, A. Yin, J.-F. Xu, Y.-H. Dong, Z.-Q. Kang, Late Cenozoic magmatic inflation, crustal thickening, and & >2 km of surface uplift in central Tibet. Geology 46, 19–22 (2018).

[51] L. Ding, P. Kapp, Y. Yue, Q. Lai, Postcollisional calc-alkaline lavas and xenoliths from the southern Qiangtang terrane, central Tibet. Earth Planet. Sci. Lett. 254, 28–38 (2007).

[52] P. Kapp, A. Yin, T. M. Harrison, L. Ding, Cretaceous-Tertiary shortening, basin development, and volcanism in central Tibet. Geol. Soc. Am. Bull. 117, 865–878 (2005).

[53] J. Meng, R. S. Coe, C. Wang, S. A. Gilder, X. Zhao, H. Liu, Y. Li, P. Ma, K. Shi, S. Li, Reduced convergence within the Tibetan Plateau by 26 Ma? Geophys. Res. Lett. 44, 6624–6632 (2017).

[54] P. J. Valdes, D. Lin, A. Farnsworth, R. A. Spicer, S. Li, S. Tao, Comment on "Revised paleoaltimetry data show low Tibetan Plateau elevation during the Eocene". Science 365, eaax8474 (2019). 31604210 10.1126/science.aax8474

[55] G. D. Hoke, Geochronology transforms our view of how Tibet’s southeast margin evolved. Geology 46, 95–96 (2018).

[56] R. A. Spicer, A. Farnsworth, T. Su, Cenozoic topography, monsoons and biodiversity conservation within the Tibetan Region: An evolving story. Plant Divers. 42, 229–254 (2020). 33094197 10.1016/j.pld.2020.06.011 PMC7567768

[57] J.-C. Zhao, A study on the structural characters of the Lunpola basin in Tibet, thesis, Chengdu University of Technology, Chengdu (2011).

[58] M. Wiedenbeck, P. Allé, F. Corfu, W. L. Griffin, M. Meier, F. Oberli, A. Von Quadt, J. C. Roddick, W. Spiegel, Three natural zircon standards for U-Th-Pb, Lu-Hf, trace element and REE analyses. Geostandard. Newslett. 19, 1–23 (1995).

[59] S. E. Jackson, N. J. Pearson, W. L. Griffin, E. A. Belousova, The application of laser ablation-inductively coupled plasma-mass spectrometry to in situ U-Pb zircon geochronology. Chem. Geol. 211, 47–69 (2004).

[60] T. E. Cerling, J. Quade, Stable carbon and oxygen isotopes in soil carbonates, in *Climate Change in Continental Isotopic Records* (American Geophysical Union, 1993), pp. 217–231.

[61] T. B. Coplen, C. Kendall, J. Hopple, Comparison of stable isotope reference samples. Nature 302, 236–238 (1983).

[62] P. Ghosh, C. N. Garzione, J. M. Eiler, Rapid uplift of the Altiplano revealed through ^13^C-^18^O bonds in paleosol carbonates. Science 311, 511–515 (2006). 16439658 10.1126/science.1119365

[63] K. J. Dennis, H. P. Affek, B. H. Passey, D. P. Schrag, J. M. Eiler, Defining an absolute reference frame for ‘clumped’ isotope studies of CO_2_. Geochim. Cosmochim. Acta 75, 7117–7131 (2011).

[64] B. Chang, W. F. Defliese, C. Li, J.-H. Huang, A. Tripati, T. J. Algeo, Effects of different constants and standards on the reproducibility of carbonate clumped isotope (Δ_47_) measurements: Insights from a long-term dataset. Rapid Commun. Mass Spectrom. 34, e8678 (2020). 31814194 10.1002/rcm.8678

[65] P. J. Valdes, E. Armstrong, M. P. S. Badger, C. D. Bradshaw, F. Bragg, M. Crucifix, T. Davies-Barnard, J. J. Day, A. Farnsworth, C. Gordon, P. O. Hopcroft, A. T. Kennedy, N. S. Lord, D. J. Lunt, A. Marzocchi, L. M. Parry, V. Pope, W. H. G. Roberts, E. J. Stone, G. J. L. Tourte, J. H. T. Williams, The BRIDGE HadCM3 family of climate models: HadCM3@Bristol v1.0. Geosci. Model Dev. 10, 3715–3743 (2017).

[66] G. L. Foster, D. L. Royer, D. J. Lunt, Future climate forcing potentially without precedent in the last 420 million years. Nat. Commun. 8, 14845 (2017). 28375201 10.1038/ncomms14845 PMC5382278

[67] D. J. Lunt, A. Farnsworth, C. Loptson, G. L. Foster, P. Markwick, C. L. O’Brien, R. D. Pancost, S. A. Robinson, N. Wrobel, Palaeogeographic controls on climate and proxy interpretation. Clim. Past 12, 1181–1198 (2016).

[68] A. Farnsworth, D. J. Lunt, C. L. O’Brien, G. L. Foster, G. N. Inglis, P. Markwick, R. D. Pancost, S. A. Robinson, Climate sensitivity on geological timescales controlled by nonlinear feedbacks and ocean circulation. Geophys. Res. Lett. 46, 9880–9889 (2019).

[69] J. Tindall, R. Flecker, P. J. Valdes, D. N. Schmidt, P. Markwick, J. Harris, Modelling the oxygen isotope distribution of ancient seawater using a coupled ocean-atmosphere GCM: Implications for reconstructing early Eocene climate. Earth Planet. Sci. Lett. 292, 265–273 (2010).

[70] R. Davies-Jones, An efficient and accurate method for computing the wet-bulb temperature along pseudoadiabats. Mon. Weather Rev. 136, 2764–2785 (2008).

[71] J. R. Buzan, K. Oleson, M. Huber, Implementation and comparison of a suite of heat stress metrics within the Community Land Model version 4.5. Geosci. Model Dev. 8, 151–170 (2015).

[72] X.-H. Liu, “Paleoelevation history and evolution of the Cenozoic Lunpola basin, central Tibet,” thesis, Institute of Tibetan Plateau Research, Chinese Academy of Sciences, Beijing (2018).

[73] G. M. Friedman, J. E. Sanders, *Principles of Sedimentology* (Wiley, 1978).

[74] D. A. Stolper, J. M. Eiler, The kinetics of solid-state isotope-exchange reactions for clumped isotopes: A study of inorganic calcites and apatites from natural and experimental samples. Am. J. Sci. 315, 363–411 (2015).

