# Supplementary material for: The rise and demise of the Paleogene Central Tibetan Valley
Source: Sci Adv. 2022 Feb 9;8(6):eabj0944. doi: 10.1126/sciadv.abj0944 (PMC8827648; doi:10.1126/sciadv.abj0944)
Supplement: Supplementary file 1 — Supplementary Materials Figs. S1 to S11 Legends for tables S1 to S5 References [file sciadv.abj0944_sm.pdf]

Science Advances
AAAS

Supplementary Materials for

The rise and demise of the Paleogene Central Tibetan Valley

Zhongyu Xiong, Xiaohui Liu, Lin Ding*, Alex Farnsworth, Robert A. Spicer, Qiang Xu, Paul Valdes, Songlin He, Deng Zeng, Chao Wang, Zhenyu Li, Xudong Guo, Tao Su, Chenyuan Zhao, Houqi Wang, Yahui Yue

*Corresponding author. Email: dinglin@itpcas.ac.cn

Published 9 February 2022, *Sci. Adv.* **8**, eabj0944 (2022)
DOI: 10.1126/sciadv.abj0944

The PDF file includes:

Supplementary Materials
Figs. S1 to S11
Legends for tables S1 to S5
References

Other Supplementary Material for this manuscript includes the following:

Tables S1 to S5

1. Detailed sedimentology

We mapped Chebuli, Dayu, Dawei and Lunpori sections for detailed sedimentology of the Niubao (E_2n) and Dingqing (E_3-N_1d) formations (Fig. S2).

The Niubao Formation is divided into three members, the lower, middle and upper members (i. e. E_2n^1, E_2n^2, E_2n^3), respectively (*72*). The lower member of the Niubao Formation (E_2n^1) is measured at both Chebuli, Dayu and Dawei sections (Figs. S2A-C), and is primarily composed of two subunits. The lower part of E_2n^1 consists of red conglomerates and sandstones with intercalations of paleosols and lenticular sandstones. The conglomerates are generally ~50-100 cm thick, well sorted, clast-supported by pebbly to cobbly metamorphic, mylonitic and volcanic clasts and show observable imbrications. The sandstone beds are generally 10-120 cm thick and medium- to coarse-grained with cross-bedding structure. Carbonate nodules were present within paleosols (Figs. S3A-B; *72*). The upper part of the E_2n^1 consists of thick brown-red mudstones interbedded with thin greyish green shale and yellow fined grained sandstones. In the upper part of E_2n^1, we discovered one tuff layer (2017TX15, Fig. S3C), which accurately constrain the upper limit of the E_2n^1, and the tuff layer is just in the lower part of the fossil site DY1 in (*17*). The lower member of Niubao Formation is interpreted to have been deposited in fluvial environments (*73*).

The middle member of the Niubao Formation (E_2n^2) was measured at the Dayu and Dawei sections (Figs. S2B-C), and is dominated by grey to greyish-green to greyish-white mudstones, siltstones and marls, interbedded with several layers of shales

and sandstones. The mudstones are laminated calcareous mudstones usually about 3-10 cm thick (*72*). Asymmetric ripple marks are sometimes preserved in the upper parts of the sandstones. The marls and shales are laminated with thicknesses of 5-10 cm and are laterally continuous for more than 500 m with prolific faunal and floral fossils preserved in the shales (*17, 30*). We found three tuff layers (2017TX17, 19, 20, Figs. S3D-F) within E_2n^2, located in the upper part of the fossil site DY2 in (*17*). Combined with the tuff layers from the E_2n^1, the age of the fossil layers can be accurately constrained. Based on the field-observed sedimentary succession, we interpret that E_2n^2 was deposited in a lacustrine system (*73*).

The upper member of the Niubao Formation (E_2n^3) was measured to the south of the Dayu Thrust at Dayu and Dawei sections (Figs. S2B-C). The strata consist of reddish conglomerates with pebbly to medium-grained sandstones and siltstones. Well-developed calcic paleosols were found at the top of several silty-mudstones (Figs. S5A-C). The strata show upward-fining rhythmicity from conglomerate to siltstone. The conglomerate, with a thickness of 2-3 m, is usually interbedded with sandstones (Fig. S5C). The sedimentary environment of E_2n^3 is interpreted as fluvial (*73*).

The Dingqing Formation (E_3-N_1d) can also be further divided into lower, middle and upper members (i. e. E_3-N_1d^1, E_3-N_1d^2, E_3-N_1d^3) according to the lithological association at Chebuli and Lunpori sections (Figs. S2A, S2D; *72*). E_3-N_1d^1 was measured at the Chebuli section (Fig. S2A), containing 1-10 cm thick grey mudstones and marls, intercalated with thin layers of grayish yellow sandstone and siltstones. A

tuff layer (2015TL45) was discovered in the lower part of the section (Fig. S5D). E_3-N_1d^2 consists of grey mudstone, shale interbedded with oil shale, siltstone and fine-grained sandstone (Fig. S2D). One tuff (2013TX76) was collected from E_3-N_1d^2 (Fig. S5E) at the Lunpori section. E_3-N_1d^3 is dominated by grey mudstone, alternating with shales, siltstones and marls (Fig. S2D). A mammalian fossil was collected from a mudstone within E_3-N_1d^3 (*28*), and we discovered two tuff layers (2017TX59, 60; Fig. S5F) in E_3-N_1d^1 near the Dayu area, which accurately constrains its sedimentary age. The lithofacies associations of the Dingqing Formation indicate a depositional environment of a medium/deep-water lake (*73*).

2. Diagenesis

Diagenesis after sedimentation will change the $\delta^{18}O_c$ signals of carbonates, and thus render inaccurate the use of isotope proxies to reconstruct paleoelevation. We employed detailed petrographic analysis and carbonate clumped isotope temperatures of paleosol nodules and marls to evaluate the potential effects of diagenetic alteration.

Recrystallization to spar is the first clear sign of diagenesis, and the primary texture of unaltered carbonate is mostly mictitic to micro-sparitic. Thin sections of carbonate samples from the Chebuli and Dayu area reveal dominantly micritic to microsparitic structure with rare penetrative calcite veins (Fig. S6), thus indicating that the carbonate samples have not undergone visible recrystallization.

The carbonate clumped isotope thermometry results are used to further identify microscale recrystallization and solid-state bond reordering on carbonate samples. All

the samples measured show T (Δ_{47})s below 40 °C, and so within the range of the T(Δ_{47})s observed for the original modern/Holocene pedogenic carbonates in Tibet (*42,74*).

3. Figures S1 to S11

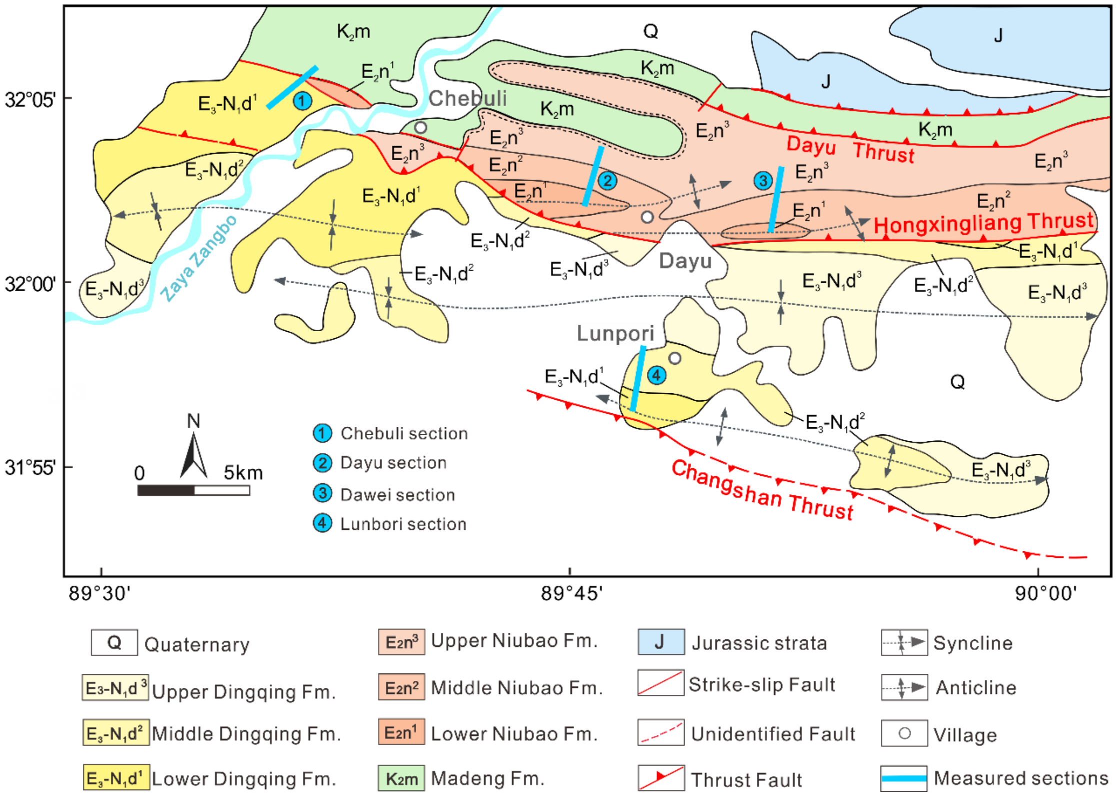

Fig. S1. Geologic map of the Lunpola Basin with the locations of the four measured sections.

The exact localities are denoted as filled blue rectangles.

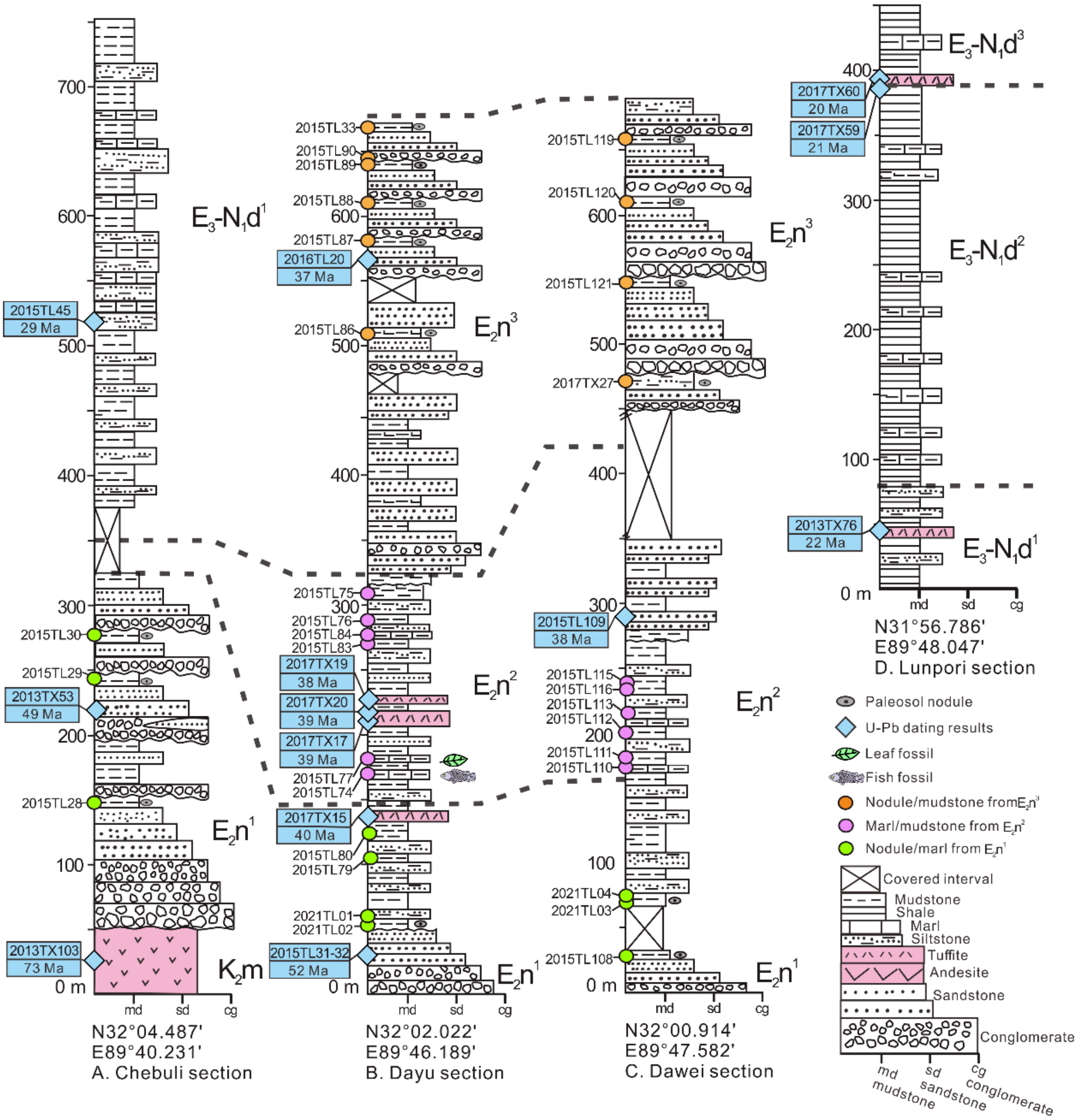

Fig. S2. Stratigraphy of the four measured sections in the Lunpola Basin. Dashed lines show lithologic correlation among these sections. Locations of carbonate samples used for isotope analyses from the lower to upper members of the Niubao Formation are marked with light green, pink and orange circles. U-Pb ages of volcanic rocks and sandstones are marked by blue diamonds. (**A**) Chebuli section with mainly the lower member of the Niubao Formation and lower member of the Dingqing Formation. (**B**) Dayu section with the Niubao Formation. (**C**) Dawei Section with the Niubao Formation. (**D**) Lunpori section with mainly the Dingqing Formation.

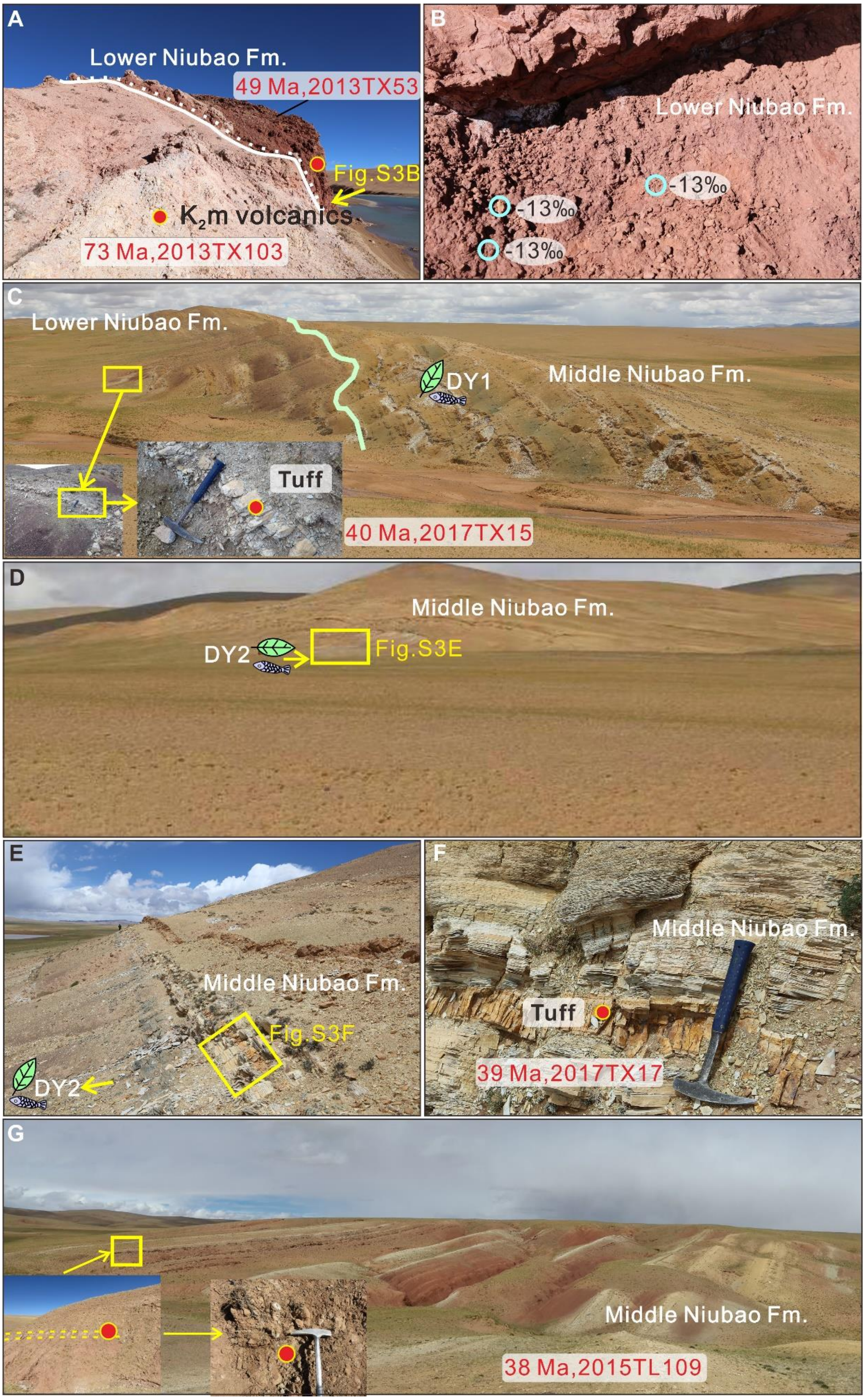

Fig. S3. Representative field photos of the lower and middle members of the Niubao Formation

(E_2n^1 and E_2n^2). (**A**) The unconformity between the early Eocene E_2n^1 and the Late Cretaceous Madeng volcanics (K_2m). (**B**) Paleosol nodules developed in E_2n^1. (**C**) The sedimentary boundary between E_2n^1 and E_2n^2. Volcanic tuff sample (2017TX15) collected from E_2n^1 with a U-Pb age of 40 Ma, which is located just below the palm and fish fossils site DY1 of (17). (**D**) The tuff sample (2017TX17) collected from the lower part of E_2n^2. This sample site locates in the upper part of the palm and fish fossils site DY2 of (17). (**E**) The tuff sample (2017TX17) collected from the lower part of E_2n^2 with a U-Pb age of 39 Ma. (**F**) Close view of the tuff sample 2017TX17. (**G**) Interbedded mudstones and marls with several layers of sandstone from E_2n^2. The inserted photo indicates the collected fine-grained sandstone samples (2015TL109) with the youngest age of 38 Ma. Photo Credit: L. Ding, ITPCAS.

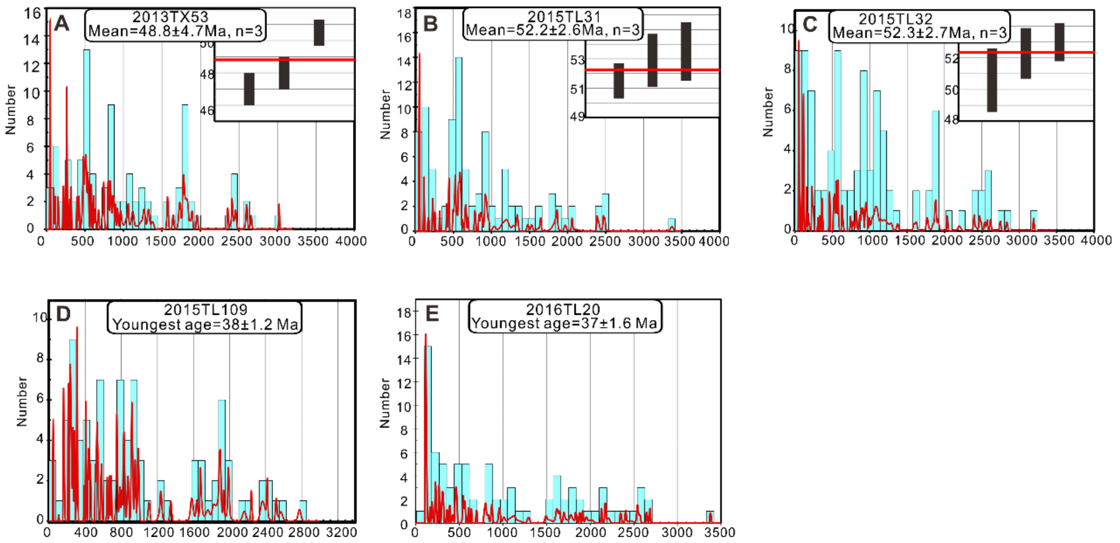

Fig. S4. Zircon U-Pb ages of sandstones from the Niubao Formation. Results are shown with probability density and the youngest zircon ages. (**A**) Sandstone (2013TX53) from E_2n^1 at the Chebuli section. (**B**) Sandstone(2015TL31) from E_2n^1 near the Dayu section. (**C**) Sandstone(2015TL32) from E_2n^1 near the Dayu section. (**D**) Sandstone(2015TL109) from E_2n^2 at the Dawei section. (**E**) Sandstone (2016TL20) from E_2n^3 at the Dayu section.

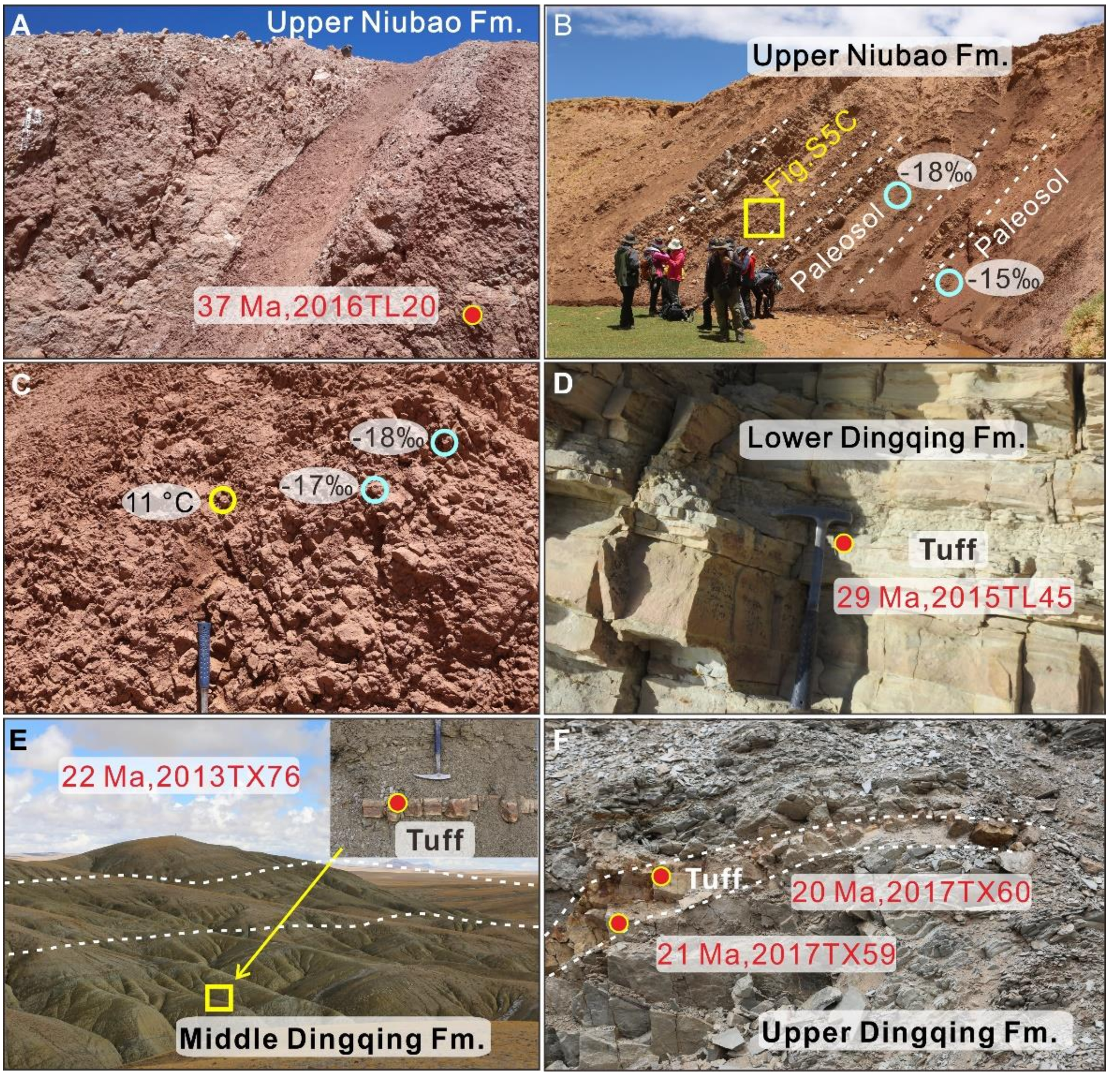

Fig. S5. Representative field photos of the upper member of the Niubao Formation (E_2n^3) and the Dingqing Formation (E_3-N_1d). (**A**) Sandstone (2016TL20) with the youngest age of 37 Ma collected from the upper most part of E_2n^3. (**B**) paleosol developed within E_2n^3 with oxygen isotope values showing in the picture. (**C**) Paleosol nodules collected from E_2n^3 with T (Δ_{47}) and oxygen isotope values showing in the picture. (**D**) The tuff layer (2015TL45) collected from E_3-N_1d^1 with U-Pb Concordia age of 29 Ma. (**E**) Interbedded marls, mudstones and siltstones of E_3-N_1d^2. The inserted layer indicates the tuff layer (2017TX76) collected from E_3-N_1d^2 with a U-Pb Concordia age of 22 Ma. (**F**) The tuff layer (2017TX59, 60) collected from E_3-N_1d^3 in the Dayu area with a U-Pb Concordia age of 20 Ma. Photo Credit: L. Ding, ITPCAS.

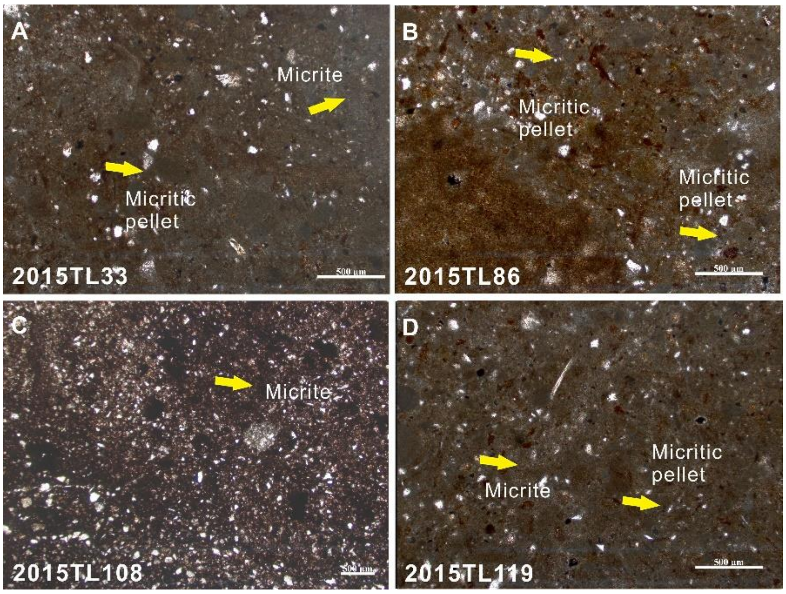

Fig. S6. Petrographic photos of the representative carbonate paleosol samples. The samples mainly show micritic structure. (**A**) 2015TL33. (**B**) 2015TL86. (**C**) 2015TL108. (**D**) 2015TL119.

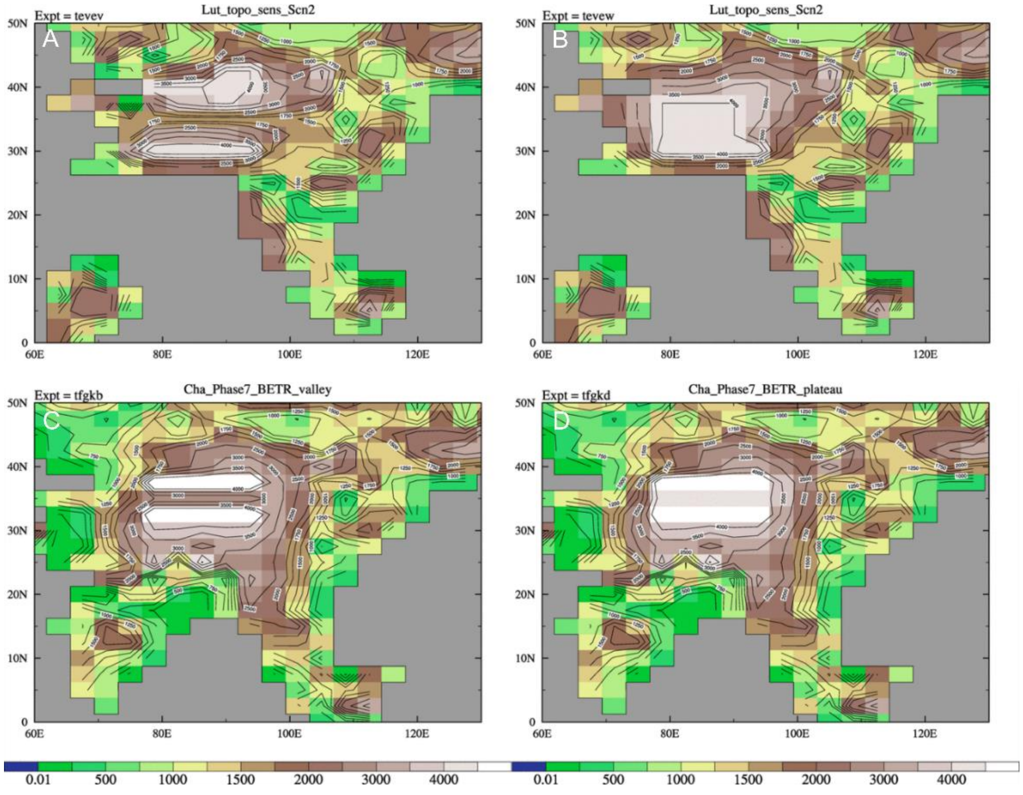

Fig. S7. Four topographic scenarios (in m) used in climate modelling. (**A**) Lutetian (~44 Ma) Tibet with a central valley at 1.5 km surrounded by 4.5 km Gangdese mountains to the south and the 4 km Central Watershed mountains to the north. (**B**) Lutetian (~44 Ma) Tibet where the central part is elevated to 4.0 km, the Gangdese mountains at 4.5 km to the south and the Central Watershed mountains of 4 km to the north. (**C**) Chattian (~25 Ma) Tibet with a low central valley at 2.5 km, straddled by the 5 km Gangdese and Central Watershed mountains to the south and north, respectively. (**D**) Chattian (~25 Ma) Tibet with a high elevated central valley at 4.5 km, straddled by 5 km Gangdese and Central Watershed

mountains to the south and north, respectively.

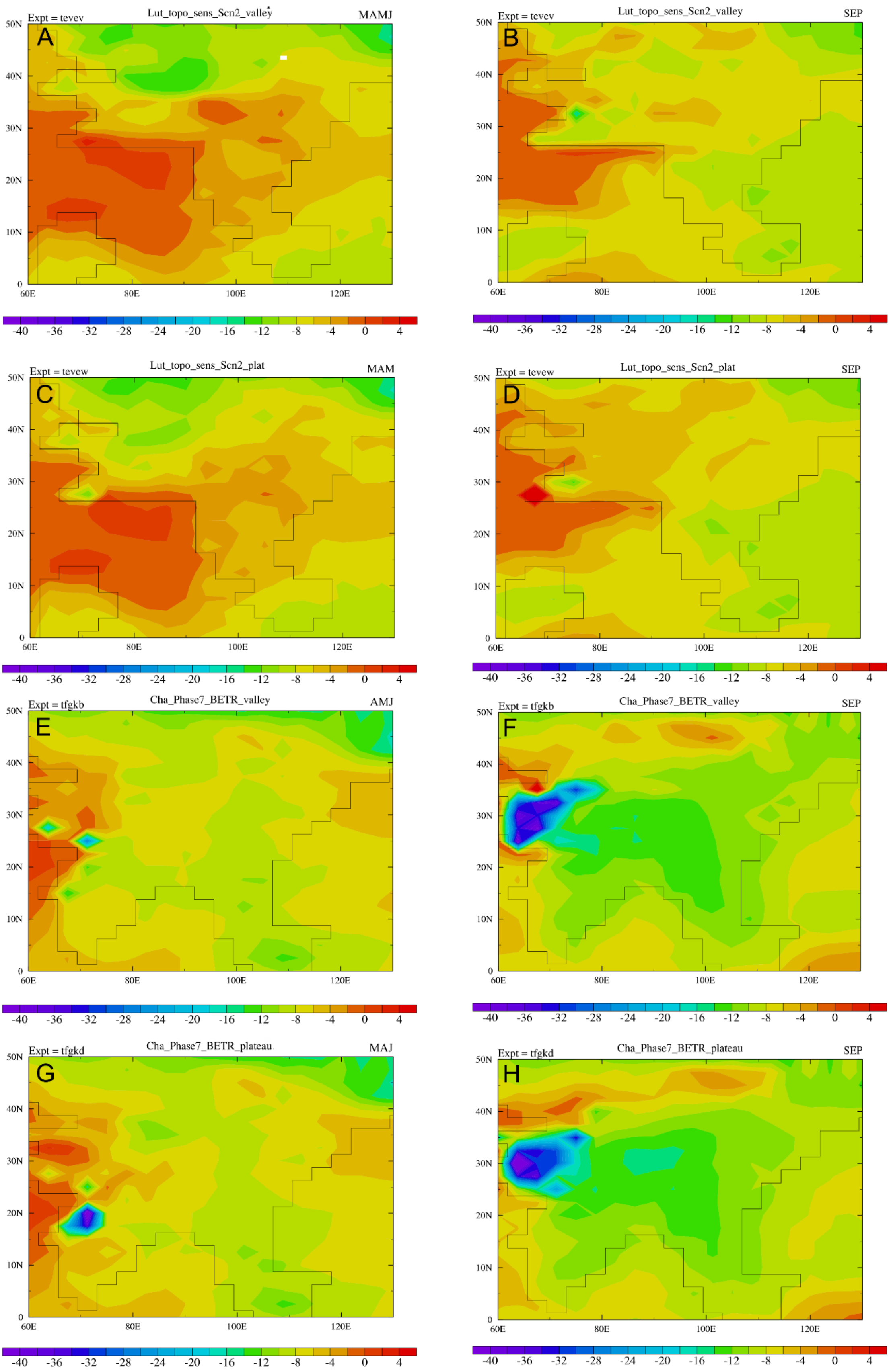

Fig. S8. $\delta^{18}O$ values (in ‰) during carbonate formation periods in four different scenarios. (**A**) $\delta^{18}O$ value during March-June for a Lutetian valley system (topographic scenario 1; CO_2 concentration 1120 ppm); (**B**) $\delta^{18}O$ value during September for a Lutetian valley system (topographic scenario 1; CO_2 concentration 1120 ppm); (**C**) $\delta^{18}O$ value during March-May for a Lutetian plateau (topographic scenario 2; CO_2 concentration 1120 ppm); (**D**) $\delta^{18}O$ value during September for a Lutetian plateau (topographic scenario 2; CO_2 concentration 1120 ppm); (**E**) $\delta^{18}O$ value during April-June for a Chattian valley system (topographic scenario 3; CO_2 concentration 560 ppm); (**F**) $\delta^{18}O$ value during September for a Chattian valley system (topographic scenario 3; CO_2 concentration 560 ppm); (**G**) $\delta^{18}O$ value during May-June for a Chattian plateau (topographic scenario 4; CO_2 concentration 560 ppm); (**H**) $\delta^{18}O$ value during September for a Chattian plateau (topographic scenario 4; CO_2 concentration 560 ppm).

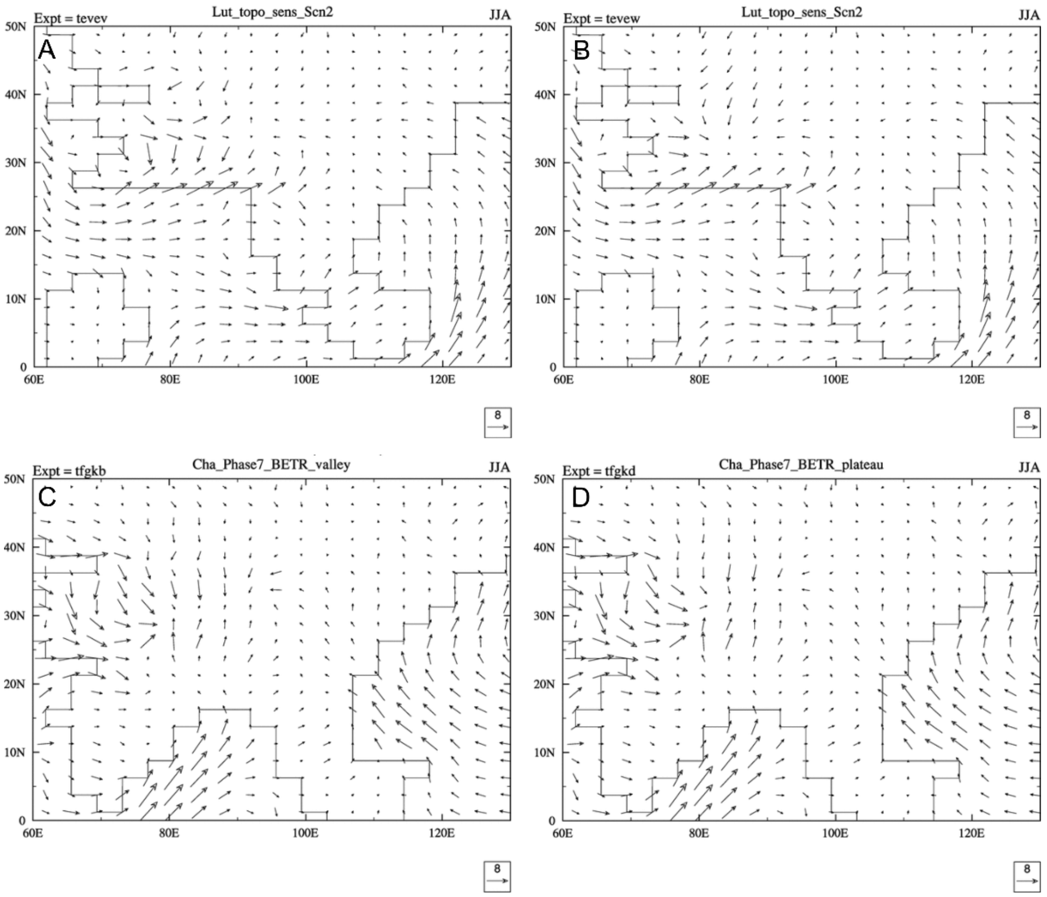

Fig S9. Surface winds at 10m (in m/s) for the four different scenarios. (**A**) Lutetian valley system (topographic scenario 1; CO_2 concentration 1120 ppm); (**B**) Lutetian plateau (topographic scenario 2; CO_2 concentration 1120 ppm); (**C**) Chattian valley system (topographic scenario 3; CO_2 concentration 560 ppm); (**D**) Chattian plateau (topographic scenario 4; CO_2 concentration 560 ppm).

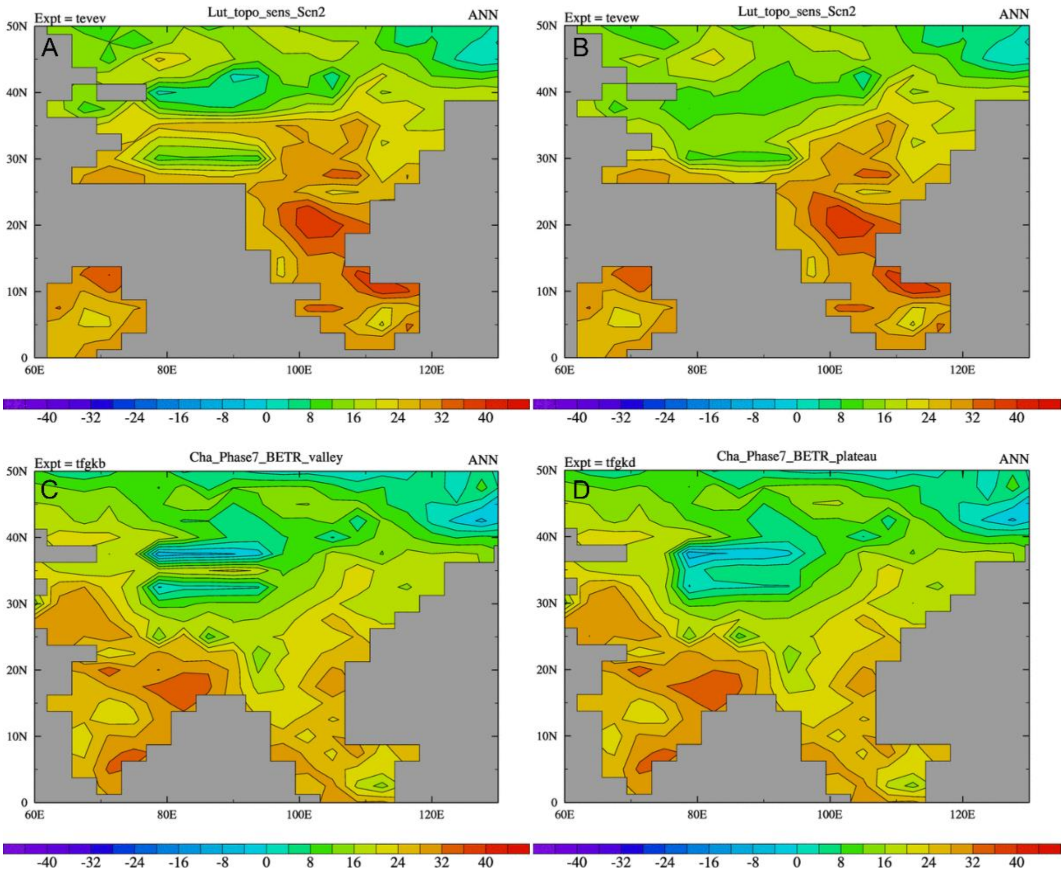

Fig. S10. 1.5 m mean annual surface air temperature results (in °C) for the four different scenarios. (**A**) Lutetian valley system (topographic scenario 1; CO_2 concentration 1120 ppm); (**B**) Lutetian plateau (topographic scenario 2; CO_2 concentration 1120 ppm); (**C**) Chattian valley system (topographic scenario 3; CO_2 concentration 560 ppm); (**D**) Chattian plateau (topographic scenario 4; CO_2 concentration 560 ppm).

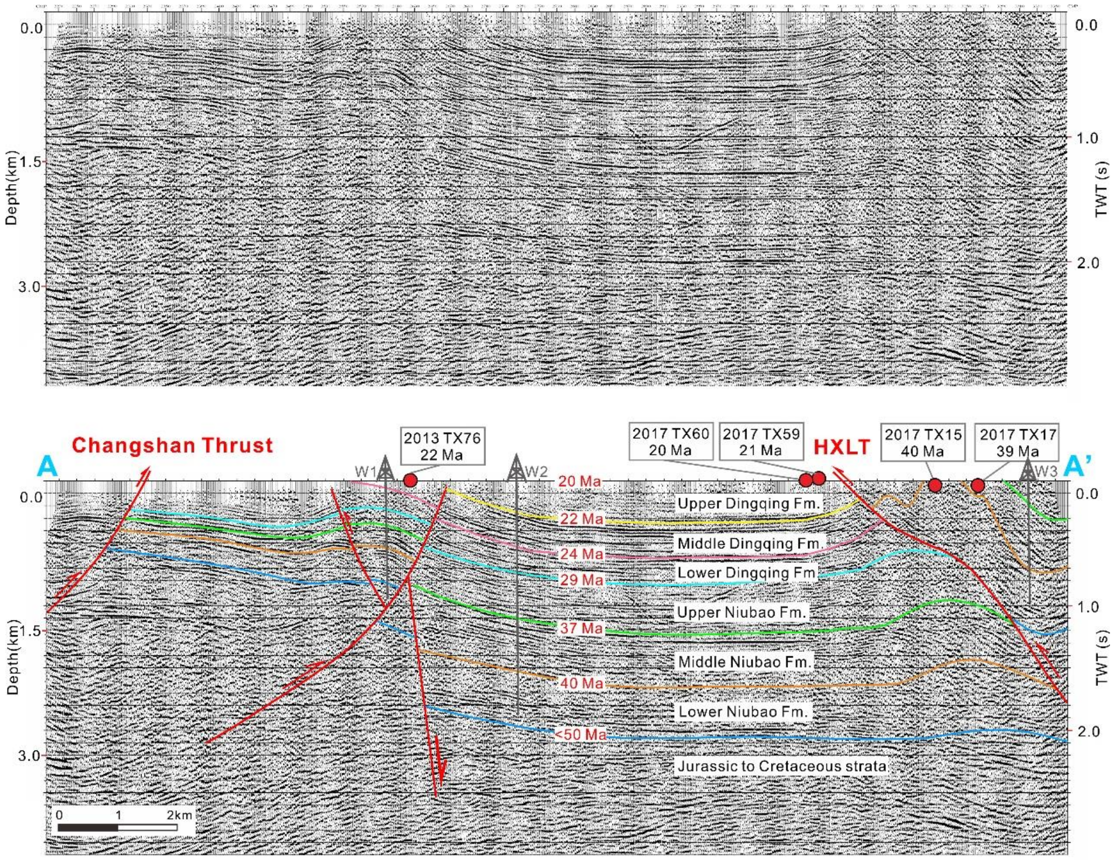

Fig. S11. Seismic profile with and without interpretations.

4. Legends for tables S1 to S5

Table S1. Zircon LA-ICP-MS U-Pb *in-situ* results for volcanic rocks and sandstones in the Lunpola Basin.

Table S2. C-O isotope results.

Table S3. Carbonate clumped isotope results.

Table S4. Model setting of the four scenarios used in the study.

Table S5. Raw clumped isotope data used in the study.

REFERENCES AND NOTES

1. A. Farnsworth, D. J. Lunt, S. A. Robinson, P. J. Valdes, W. H. G. Roberts, P. D. Clift, P. Markwick, T. Su, N. Wrobel, F. Bragg, S. Kelland, R. D. Pancost, Past East Asian monsoon evolution controlled by paleogeography, not CO_2. *Sci. Adv.* **5**, eaax1697 (2019).

2. P. Molnar, P. England, J. Martinod, Mantle dynamics, uplift of the Tibetan Plateau, and the Indian monsoon. *Rev. Geophys.* **31**, 357–396 (1993).

3. S.-F. Li, P. J. Valdes, A. Farnsworth, T. Davies-Barnard, T. Su, D. J. Lunt, R. A. Spicer, J. Liu, W.-Y.-D. Deng, J. Huang, H. Tang, A. Ridgwell, L.-L. Chen, Z.-K. Zhou, Orographic evolution of northern Tibet shaped vegetation and plant diversity in eastern Asia. *Sci. Adv.* **7**, eabc7741 (2021).

4. P. Molnar, W. R. Boos, D. S. Battisti, Orographic controls on climate and paleoclimate of Asia: Thermal and mechanical roles for the Tibetan Plateau. *Annu. Rev. Earth Planet. Sci.* **38**, 77–102 (2010).

5. T. M. Harrison, P. Copeland, W. S. F. Kidd, A. Yin, Raising Tibet. *Science* **255**, 1663–1670 (1992).

6. P. England, G. Houseman, Extension during continental convergence, with application to the Tibetan Plateau. *J. Geophys. Res.* **94**, 17561–17579 (1989).

7. L. Ding, Q. Xu, Y.-H. Yue, H.-Q. Wang, F.-L. Cai, S. Li, The Andean-type Gangdese Mountains: Paleoelevation record from the Paleocene-Eocene Linzhou Basin. *Earth Planet. Sci. Lett.* **392**, 250–264 (2014).

8. C.-S Wang, X.-X. Zhao, Z.-F. Liu, P. C. Lippert, S. A. Graham, R. S. Coe, H.-S. Yi, L.-D. Zhu, S. Liu, Y.-L. Li, Constraints on the early uplift history of the Tibetan Plateau. *Proc. Natl. Acad. Sci. U.S.A.* **105**, 4987–4992 (2008).

9. A. Mulch, C. P. Chamberlain, The rise and growth of Tibet. *Nature* **439**, 670–671 (2006).

10. L. H. Royden, B. C. Burchfiel, R. D. van der Hilst, The geological evolution of the Tibetan plateau. *Science* **321**, 1054–1058 (2008).

11. P. Tapponnier, Z.-Q. Xu, F. Roger, B. Meyer, N. Arnaud, G. Wittlinger, J.-S. Yang, Oblique stepwise rise and growth of the Tibet plateau. *Science* **294**, 1671–1677 (2001).

12. P. England, G. Houseman, Finite strain calculations of continental deformation 2. Comparison with the India-Asia collision zone. *J. Geophys. Res.* **91**, 3664–3676 (1986).

13. C.-S. Wang, J.-G. Dai, X.-X Zhao, Y.-L. Li, S. A. Graham, D.-F. He, B. Ran, J. Meng, Outward-growth of the Tibetan Plateau during the Cenozoic: A review. *Tectonics* **621**, 1–43 (2014).

14. P. Kapp, P. G. DeCelles, G. E. Gehrels, M. Heizler, L. Ding, Geological records of the Lhasa-Qiangtang and Indo-Asian collisions in the Nima area of central Tibet. *Geol. Soc. Am. Bull.* **119**, 917–933 (2007).

15. Z.-Y. Xiong, L. Ding, R. A. Spicer, A. Farnsworth, X. Wang, P. J. Valdes, T. Su, Q.-H. Zhang, L.-Y. Zhang, F.-L. Cai, H.-Q. Wang, Z.-Y. Li, P.-P. Song, X.-D. Guo, Y.-H. Yue, The early Eocene rise of the Gonjo Basin, SE Tibet: From low desert to high forest. *Earth Planet. Sci. Lett.* **543**, 116312 (2020).

16. T. Su, R. A. Spicer, F.-X. Wu, A. Farnsworth, J. Huang, C. Del Rio, T. Deng, L. Ding, W.-Y.-D. Deng, Y.-J. Huang, A. Hughes, L.-B. Jia, J.-H. Jin, S.-F. Li, S.-Q. Liang, J. Liu, X.-Y. Liu, S. Sherlock, T. Spicer, G. Srivastava, H. Tang, P. Valdes, T.-X. Wang, M. Widdowson, M.-X. Wu, Y.-W. Xing, C.-L. Xu, J. Yang, C. Zhang, S.-T. Zhang, X.-W. Zhang, F. Zhao, Z.-K. Zhou, A Middle Eocene lowland humid subtropical "Shangri-La" ecosystem in central Tibet. *Proc. Natl. Acad. Sci. U.S.A.* **117**, 32989–32995 (2020).

17. T. Su, A. Farnsworth, R. A. Spicer, J. Huang, F.-X. Wu, J. Liu, S.-F. Li, Y.-W. Xing, Y.-J. Huang, W.-Y.-D. Deng, H. Tang, C.-L. Xu, F. Zhao, G. Srivastava, P. J. Valdes, T. Deng, Z.-K. Zhou, No high Tibetan Plateau until the Neogene. *Sci. Adv.* **5**, eaav2189 (2019).

18. A. Yin, T. M. Harrison, Geologic evolution of the Himalayan-Tibetan orogen. *Annu. Rev. Earth Planet. Sci.* **28**, 211–280 (2000).

19. P. Kapp, P. G. DeCelles, Mesozoic-Cenozoic geological evolution of the Himalayan-Tibetan orogen and working tectonic hypotheses. *Am. J. Sci.* **319**, 159–254 (2019).

20. C. Chengfa, C. Nansheng, M. P. Coward, D. Wanming, J. F. Dewey, A. Gansser, N. B. W. Harris, J. Chengwei, W. S. F. Kidd, M. R. Leeder, L. Huan, L. Jinlu, L. Chengjie, M. Houjun, P. Molnar, P. Yun, P. Yusheng, J. A. Pearce, R. M. Shackleton, A. B. Smith, S. Yiyin, M. Ward, D. R. Watts, X. Juntao, X. Ronghua, Y. Jixiang, Z. Yuquan, Preliminary conclusions of the Royal Society and Academia Sinica 1985 Geotraverse of Tibet. *Nature* **323**, 501–507 (1986).

21. M. Taylor, A. Yin, F. J. Ryerson, P. Kapp, L. Ding, Conjugate strike-slip faulting along the Bangong-Nujiang suture zone accommodates coeval east-west extension and north-south shortening in the interior of the Tibetan Plateau. *Tectonics* **22**, 1044 (2003).

22. A. Yin, M. H. Taylor, Mechanics of V-shaped conjugate strike-slip faults and the corresponding continuum mode of continental deformation. *Geol. Soc. Am. Bull.* **123**,1798–1821(2011).

23. P. Kapp, M. A. Murphy, A. Yin, T. M. Harrison, L. Ding, J. Guo, Mesozoic and Cenozoic tectonic evolution of the Shiquanhe area of western Tibet. *Tectonics* **22**, 1029 (2003).

24. Y. Wei, K. Zhang, C. N. Garzione, Y. Xu, B. Song, J. Ji, Low palaeoelevation of the northern Lhasa terrane during late Eocene: Fossil foraminifera and stable isotope evidence from the Gerze Basin. *Sci. Rep.* **6**, 27508 (2016).

25. D. B. Rowley, B. S. Currie, Palaeo-altimetry of the late Eocene to Miocene Lunpola basin, central Tibet. *Nature* **439**, 677–681 (2006).

26. P. J. Polissar, K. H. Freeman, D. B. Rowley, F. A. McInerney, B. S. Currie, Paleoaltimetry of the Tibetan Plateau from *D/H* ratios of lipid biomarkers. *Earth Planet. Sci. Lett.* **287**, 64–76 (2009).

27. J. Sun, Q. Xu, W. Liu, Z. Zhang, L. Xue, P. Zhao, Palynological evidence for the latest Oligocene–early Miocene paleoelevation estimate in the Lunpola Basin, central Tibet. *Palaeogeogr. Palaeoeclimatol. Palaeoecol.* **399**, 21–30 (2014).

28. T. Deng, S. Wang, G. Xie, Q. Li, S. Hou, B. Sun, A mammalian fossil from the Dingqing Formation in the Lunpola Basin, northern Tibet, and its relevance to age and paleo-altimetry. *Chin. Sci. Bull.* **57**, 261–269 (2011).

29. G. Jia, Y. Bai, Y. Ma, J. Sun, P. Peng, Paleoelevation of Tibetan Lunpola basin in the Oligocene-Miocene transition estimated from leaf wax lipid dual isotopes. *Global Planet. Change* **126**, 14–22 (2015).

30. F. Wu, D. Miao, M.-m. Chang, G. Shi, N. Wang, Fossil climbing perch and associated plant megafossils indicate a warm and wet central Tibet during the late Oligocene. *Sci. Rep.* **7**, 878 (2017).

31. X. Fang, G. Dupont-Nivet, C. Wang, C. Song, Q. Meng, W. Zhang, J. Nie, T. Zhang, Z. Mao, Y. Chen, Revised chronology of central Tibet uplift (Lunpola Basin). *Sci. Adv.* **6**, eaba7298 (2020).

32. T. Westerhold, N. Marwan, A. J. Drury, D. Liebrand, C. Agnini, E. Anagnostou, J. S. K. Barnet, S. M. Bohaty, D. De Vleeschouwer, F. Florindo, T. Frederichs, D. A. Hodell, A. E. Holbourn, D. Kroon, V. Lauretano, K. Littler, L. J. Lourens, M. Lyle, H. Pälike, U. Röhl, J. Tian, R. H. Wilkens, P. A. Wilson, J. C. Zachos, An astronomically dated record of Earth's climate and its predictability over the last 66 million years. *Science* **369**, 1383–1387 (2020).

33. A. Farnsworth, P. J. Valdes, R. A. Spicer, L. Ding, C. Witkowski, V. Lauretano, T. Su, S. Li, S. Li, Z. Zhou, Paleoclimate model-derived thermal lapse rates: Towards increasing precision in paleoaltimetry studies. *Earth Planet. Sci. Lett.* **564**, 116903 (2021).

34. A. K. Laskowski, D. A. Orme, F.-L. Cai, L. Ding, The Ancestral Lhasa River: A Late Cretaceous trans-arc river that drained the proto–Tibetan Plateau. *Geology* **47**, 1029–1033 (2019).

35. H. He, J. Sun, Q. Li, R. Zhu, New age determination of the Cenozoic Lunpola basin, central Tibet. *Geol. Mag.* **149**, 141–145 (2012).

36. P. G. DeCelles, J. Quade, P. Kapp, M. Fan, D. L. Dettman, L. Ding, High and dry in central Tibet during the Late Oligocene. *Earth Planet. Sci. Lett.* **253**, 389–401 (2007).

37. C. N. Garzione, G. D. Hoke, J. C. Libarkin, S. Withers, B. MacFadden, J. Eiler, P. Ghosh, A. Mulch, Rise of the Andes. *Science* **320**, 1304–1307 (2008).

38. J. R. Kelson, K. W. Huntington, A. J. Schauer, C. Saenger, A. R. Lechler, Toward a universal carbonate clumped isotope calibration: Diverse synthesis and preparatory methods suggest a single temperature relationship. *Geochim. Cosmochim. Acta* **197**, 104–131 (2017).

39. H. Li, X. Liu, A. Arnold, B. Elliott, R. Flores, A. M. Kelley, A. Tripati, Mass 47 clumped isotope signatures in modern lacustrine authigenic carbonates in Western China and other regions and implications for paleotemperature and paleoelevation reconstructions. *Earth Planet. Sci. Lett.* **562**, 116840 (2021).

40. M. R. Talbot, A review of the palaeohydrological interpretation of carbon and oxygen isotopic ratios in primary lacustrine carbonates. *Chem. Geol.* **80**, 261–279 (1990).

41. J. R. Kelson, K. W. Huntington, D. O. Breecker, L. K. Burgener, T. M. Gallagher, G. D. Hoke, S. V. Petersen, A proxy for all seasons? A synthesis of clumped isotope data from Holocene soil carbonates. *Quat. Sci. Rev.* **234**, 106259 (2020).

42. J. Quade, J. Eiler, M. Daëron, H. Achyuthan, The clumped isotope geothermometer in soil and paleosol carbonate. *Geochim. Cosmochim. Acta* **105**, 92–107 (2013).

43. M. C. Ringham, G. D. Hoke, K. W. Huntington, J. N. Aranibar, Influence of vegetation type and site-to-site variability on soil carbonate clumped isotope records, Andean piedmont of Central Argentina (32–34°S). *Earth Planet. Sci. Lett.* **440**, 1–11 (2016).

44. D. O. Breecker, Z. D. Sharp, L. D. McFadden, Seasonal bias in the formation and stable isotopic composition of pedogenic carbonate in modern soils from central New Mexico, USA. *Geol. Soc. Am. Bull.* **121**, 630–640 (2009).

45. H. Lu, W. Liu, H. Yang, H. Wang, Z. Liu, Q. Leng, Y. Sun, W. Zhou, Z. An, 800-kyr land temperature variations modulated by vegetation changes on Chinese Loess Plateau. *Nat. Commun.* **10**, 1958 (2019).

46. H. Bhatia, G. Srivastava, R. A. Spicer, A. Farnsworth, T. E. V. Spicer, R. C. Mehrotra, K. N. Paudayal, P. Valdes, Leaf physiognomy records the Miocene intensification of the South Asia Monsoon. *Global Planet. Change* **196**, 103365 (2021).

47. S.-T. Kim, J. R. O'Neil, Equilibrium and nonequilibrium oxygen isotope effects in synthetic carbonates. *Geochim. Cosmochim. Acta* **61**, 3461–3475 (1997).

48. F. A. Smith, K. H. Freeman, Influence of physiology and climate on δD of leaf wax *n*-alkanes from C_3 and C_4 grasses. *Geochim. Cosmochim. Acta* **70**, 1172–1187 (2006).

49. M. Chen, F. Niu, J. Tromp, A. Lenardic, C.-T. A. Lee, W. Cao, J. Ribeiro, Lithospheric foundering and underthrusting imaged beneath Tibet. *Nat. Commun.* **8**, 15659 (2017).

50. J.-L. Chen, A. Yin, J.-F. Xu, Y.-H. Dong, Z.-Q. Kang, Late Cenozoic magmatic inflation, crustal thickening, and >2 km of surface uplift in central Tibet. *Geology* **46**, 19–22 (2018).

51. L. Ding, P. Kapp, Y. Yue, Q. Lai, Postcollisional calc-alkaline lavas and xenoliths from the southern Qiangtang terrane, central Tibet. *Earth Planet. Sci. Lett.* **254**, 28–38 (2007).

52. P. Kapp, A. Yin, T. M. Harrison, L. Ding, Cretaceous-Tertiary shortening, basin development, and volcanism in central Tibet. *Geol. Soc. Am. Bull.* **117**, 865–878 (2005).

53. J. Meng, R. S. Coe, C. Wang, S. A. Gilder, X. Zhao, H. Liu, Y. Li, P. Ma, K. Shi, S. Li, Reduced convergence within the Tibetan Plateau by 26 Ma? *Geophys. Res. Lett.* **44**, 6624–6632 (2017).

54. P. J. Valdes, D. Lin, A. Farnsworth, R. A. Spicer, S. Li, S. Tao, Comment on "Revised paleoaltimetry data show low Tibetan Plateau elevation during the Eocene". *Science* **365**, eaax8474 (2019).

55. G. D. Hoke, Geochronology transforms our view of how Tibet's southeast margin evolved. *Geology* **46**, 95–96 (2018).

56. R. A. Spicer, A. Farnsworth, T. Su, Cenozoic topography, monsoons and biodiversity conservation within the Tibetan Region: An evolving story. *Plant Divers.* **42**, 229–254 (2020).

57. J.-C. Zhao, A study on the structural characters of the Lunpola basin in Tibet, thesis, Chengdu University of Technology, Chengdu (2011).

58. M. Wiedenbeck, P. Allé, F. Corfu, W. L. Griffin, M. Meier, F. Oberli, A. Von Quadt, J. C. Roddick, W. Spiegel, Three natural zircon standards for U-Th-Pb, Lu-Hf, trace element and REE analyses. *Geostandard. Newslett.* **19**, 1–23 (1995).

59. S. E. Jackson, N. J. Pearson, W. L. Griffin, E. A. Belousova, The application of laser ablation-inductively coupled plasma-mass spectrometry to in situ U-Pb zircon geochronology. *Chem. Geol.* **211**, 47–69 (2004).

60. T. E. Cerling, J. Quade, Stable carbon and oxygen isotopes in soil carbonates, in *Climate Change in Continental Isotopic Records* (American Geophysical Union, 1993), pp. 217–231.

61. T. B. Coplen, C. Kendall, J. Hopple, Comparison of stable isotope reference samples. *Nature* **302**, 236–238 (1983).

62. P. Ghosh, C. N. Garzione, J. M. Eiler, Rapid uplift of the Altiplano revealed through ^{13}C-^{18}O bonds in paleosol carbonates. *Science* **311**, 511–515 (2006).

63. K. J. Dennis, H. P. Affek, B. H. Passey, D. P. Schrag, J. M. Eiler, Defining an absolute reference frame for 'clumped' isotope studies of CO_2. *Geochim. Cosmochim. Acta* **75**, 7117–7131 (2011).

64. B. Chang, W. F. Defliese, C. Li, J.-H. Huang, A. Tripati, T. J. Algeo, Effects of different constants and standards on the reproducibility of carbonate clumped isotope (Δ_{47}) measurements: Insights from a long-term dataset. *Rapid Commun. Mass Spectrom.* **34**, e8678 (2020).

65. P. J. Valdes, E. Armstrong, M. P. S. Badger, C. D. Bradshaw, F. Bragg, M. Crucifix, T. Davies-Barnard, J. J. Day, A. Farnsworth, C. Gordon, P. O. Hopcroft, A. T. Kennedy, N. S. Lord, D. J. Lunt, A. Marzocchi, L. M. Parry, V. Pope, W. H. G. Roberts, E. J. Stone, G. J. L. Tourte, J. H. T. Williams, The BRIDGE HadCM3 family of climate models: HadCM3@Bristol v1.0. *Geosci. Model Dev.* **10**, 3715–3743 (2017).

66. G. L. Foster, D. L. Royer, D. J. Lunt, Future climate forcing potentially without precedent in the last 420 million years. *Nat. Commun.* **8**, 14845 (2017).

67. D. J. Lunt, A. Farnsworth, C. Loptson, G. L. Foster, P. Markwick, C. L. O'Brien, R. D. Pancost, S. A. Robinson, N. Wrobel, Palaeogeographic controls on climate and proxy interpretation. *Clim. Past* **12**, 1181–1198 (2016).

68. A. Farnsworth, D. J. Lunt, C. L. O'Brien, G. L. Foster, G. N. Inglis, P. Markwick, R. D. Pancost, S. A. Robinson, Climate sensitivity on geological timescales controlled by nonlinear feedbacks and ocean circulation. *Geophys. Res. Lett.* **46**, 9880–9889 (2019).

69. J. Tindall, R. Flecker, P. J. Valdes, D. N. Schmidt, P. Markwick, J. Harris, Modelling the oxygen isotope distribution of ancient seawater using a coupled ocean-atmosphere GCM: Implications for reconstructing early Eocene climate. *Earth Planet. Sci. Lett.* **292**, 265–273 (2010).

70. R. Davies-Jones, An efficient and accurate method for computing the wet-bulb temperature along pseudoadiabats. *Mon. Weather Rev.* **136**, 2764–2785 (2008).

71. J. R. Buzan, K. Oleson, M. Huber, Implementation and comparison of a suite of heat stress metrics within the Community Land Model version 4.5. *Geosci. Model Dev.* **8**, 151–170 (2015).

72. X.-H. Liu, "Paleoelevation history and evolution of the Cenozoic Lunpola basin, central Tibet," thesis, Institute of Tibetan Plateau Research, Chinese Academy of Sciences, Beijing (2018).

73. G. M. Friedman, J. E. Sanders, *Principles of Sedimentology* (Wiley, 1978).

74. D. A. Stolper, J. M. Eiler, The kinetics of solid-state isotope-exchange reactions for clumped isotopes: A study of inorganic calcites and apatites from natural and experimental samples. *Am. J. Sci.* **315**, 363–411 (2015).
